# Nitric Oxide Photo-Donor Hybrids of Ciprofloxacin and Norfloxacin: A Shift in Activity from Antimicrobial to Anticancer Agents

**DOI:** 10.1021/acs.jmedchem.1c00917

**Published:** 2021-07-28

**Authors:** Antonino Nicolò Fallica, Carla Barbaraci, Emanuele Amata, Lorella Pasquinucci, Rita Turnaturi, Maria Dichiara, Sebastiano Intagliata, Marzia Bruna Gariboldi, Emanuela Marras, Viviana Teresa Orlandi, Claudia Ferroni, Cecilia Martini, Antonio Rescifina, Davide Gentile, Greta Varchi, Agostino Marrazzo

**Affiliations:** † Department of Drug and Health Sciences (DSFS), University of Catania, Viale A. Doria, 6, 95125 Catania, Italy; ‡ Department of Biotechnology and Life Sciences (DBSV), University of Insubria, Via JH Dunant 3, 21100 Varese, Italy; § Institute for the Organic Synthesis and Photoreactivity − ISOF, Via Piero Gobetti, 101, 40129 Bologna, Italy

## Abstract

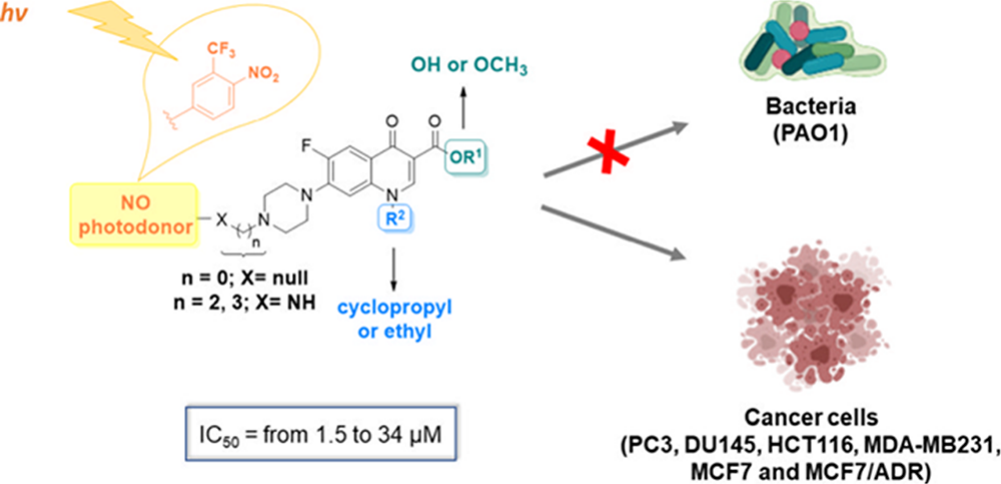

The potential anticancer effect of fluoroquinolone antibiotics has been recently unveiled and related to their ability to interfere with DNA topoisomerase II. We herein envisioned the design and synthesis of novel Ciprofloxacin and Norfloxacin nitric oxide (NO) photo-donor hybrids to explore the potential synergistic antitumor effect exerted by the fluoroquinolone scaffold and NO eventually produced upon light irradiation. Anticancer activity, evaluated on a panel of tumor cell lines, showed encouraging results with IC_50_ values in the low micromolar range. Some compounds displayed intense antiproliferative activity on triple-negative and doxorubicin-resistant breast cancer cell lines, paving the way for their potential use to treat aggressive, refractory and multidrug-resistant breast cancer. No significant additive effect was observed on PC3 and DU145 cells following NO release. Conversely, antimicrobial photodynamic experiments on both Gram-negative and Gram-positive microorganisms displayed a significant killing rate in *Staphylococcus aureus*, accounting for their potential effectiveness as selective antimicrobial photosensitizers.

## Introduction

Since their early discovery as byproducts of chloroquine synthesis,^1^ quinolones have represented one of the most important classes of antibiotics for urinary and respiratory infection treatment.^2−4^ Quinolones exert their bactericidal activity by interfering with DNA gyrase in Gram-negative bacteria and topoisomerase IV in Gram-positive bacteria.^5^ Both enzymes belong to the topoisomerase family, which plays an essential role in the regulation of the DNA topological state, in DNA replication, and in the condensation and segregation of chromosomes.^6,7^ In the presence of quinolone, the enzyme forms a DNA/enzyme/drug ternary complex that perturbs DNA replication, leading to bacterial death or eukaryotic cell apoptosis.

**Figure 1:**
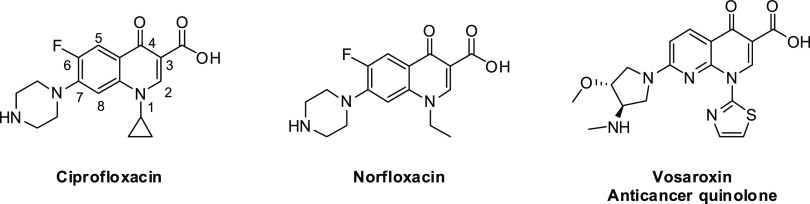
Chemical structures of Ciprofloxacin, Norfloxacin, and Vosaroxin.

Structural modifications of the first marketed compound of this class of molecules, nalidixic acid, generated compounds with greater potency, a broader spectrum of activity, improved pharmacokinetics, and lower frequency of acquired resistance. In particular, Norfloxacin (Nor) and Ciprofloxacin (Cip), belonging to the second-generation fluoroquinolones (Figure 1), displayed increased potency and affinity for Gram-negative bacteria due to the introduction of a fluorine atom at position 6. Subsequent structure modifications led to third- and fourth-generation fluoroquinolones, with improved efficacy against Gram-positive organisms.^2,8,9^ Also, structure–activity relationship (SAR) studies highlighted the importance of the *N-*1 substituent on the quinolone core together with the presence of either a carboxylic acid function in position 3 and a ketone function in position 4. Furthermore, to expand their spectrum of action and improve pharmacokinetics, a saturated heterocyclic ring containing an amine function was introduced at the 7-position from the second-generation fluoroquinolones.^10^

Of note, recent evidence indicates that higher doses of these drugs exert anticancer effects,^11,12^ and as expected, this effect is related to their ability to interfere with DNA topoisomerase II, the gyrase human counterpart, which is a well-known target of several anticancer drugs, such as epipodophyllotoxins (etoposide), anthracyclines (doxorubicin and daunorubicin), amsacrine, and mitoxantrone. In this view, we envisioned the design and synthesis of a novel class of 4-quinolone-based topoisomerase II inhibitors endowing the enhancement of their cytotoxic activity.

**Figure 2:**
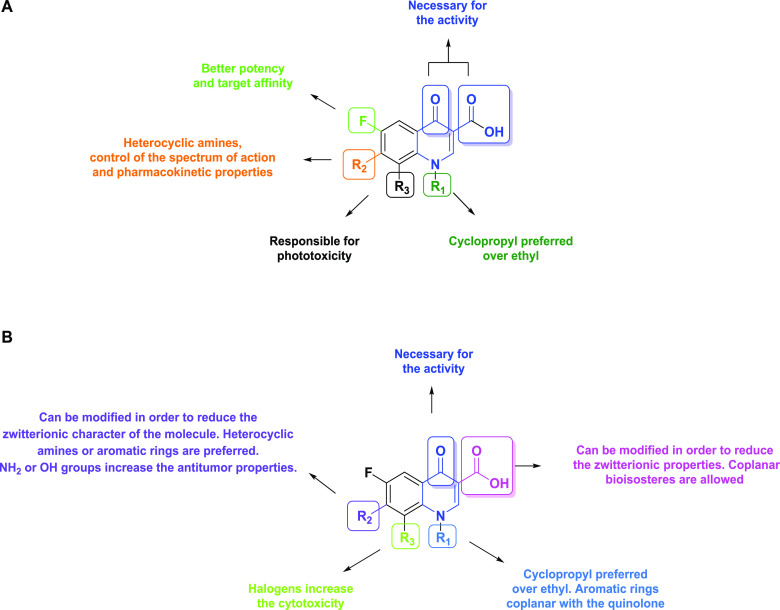
Antimicrobial (A) and antitumoral (B) structure–activity relationships of 4-quinolones.

SAR studies on anticancer quinolones (Figure 2A,B)^4,13−15^ allowed identifying the types of structure modifications boosting anticancer activity, such as the reduction of their zwitterionic character by modifying the carboxylic group at the 3-position or by adding a proper substituent on the aliphatic heterocyclic amine at the 7-position. Out of these studies, Vosaroxin^16^ has reached phase III clinical trial investigation (Figure 1).^17^ Interestingly, Vosaroxin, such as other molecules of the same class, appears to be devoid of the typical side effects of topoisomerase II inhibitors, namely, significant cardiotoxicity and cross-resistance with other topoisomerase II inhibitors, while preserving the cytotoxic effect in multidrug-resistant (MDR) or inactivated p53 cell lines.^13^

The combination of different therapeutic functionalities with synergic or additive features within the same molecular scaffold represents a fascinating opportunity to improve the overall treatment efficacy. Furthermore, the possibility of administering a single drug bearing multiple biological “effectors” could allow higher control on pharmacokinetic and side effects while boosting the treatment outcome.^18−22^

In this context, the use of molecules able to release nitric oxide (NO) under the exclusive control of light, *e*.*g*., NO photo-donors, has been recently described for antibacterial and anticancer treatment.^23,24^ NO is physiologically produced by the NO synthase enzyme family from l-arginine and O_2_^25^ and performs multiple physiological roles ranging from the control of the vascular tone to neurotransmission.^26,27^ Moreover, NO possesses antimicrobial properties^28^ and has been shown to be a key player in cancer biology, where its role seems to be regulated by several factors, such as the tumor cell subtype, NO cell sensitivity, exposure time, and cellular concentration.^29^ In fact, unlike pico- and nanomolar NO concentrations are known to boost cancer progression and invasiveness, micromolar NO concentrations promote cytotoxicity^30^ and interfere with P-glycoprotein activity,^31^ thus representing a promising option to tackle MDR phenomena. Due to the intrinsic difficulties posed by the direct administration of gaseous NO, specific NO donors,^32^ alone or included in nanomaterials, have been actively investigated.^33,34^ In particular, molecules able to release NO upon the application of an external stimulus, such as light, have attracted increasing attention due to the possibility of precisely controlling the NO production and release only at the site of interest.^35−42^

Based on the above, we herein report the design, synthesis, characterization, and molecular modeling studies of 12 Cip and Nor derivatives, endowed with a 4-nitro-3-trifluoromethyl-aniline moiety for the light-triggered release of NO. The new derivatives’ biological activity has also been evaluated, both in prokaryotic and tumor cells, along with the effect of NO release on cell viability and compared with Cip and Nor.

## Results and Discussion

### Design and Synthesis of Novel Ciprofloxacin and Norfloxacin Hybrids

The new Cip and Nor derivatives are characterized by a carboxylic or a methyl ester group at the 3-position and a NO photo-donor directly linked to the *N*-4 atom of the piperazine ring (compounds **1a**, **1b**, **3a**, and **3b**) or connected through an alkyl spacer made of two or three methylene units (compounds **6a**–**6d** and **7a**–**7d**) (Table 1).

**Table 1:**
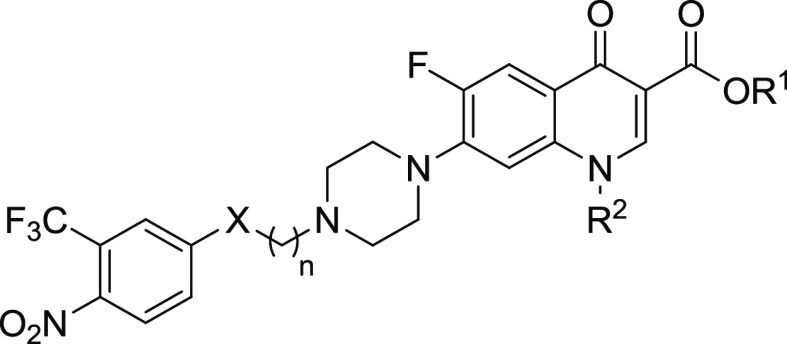

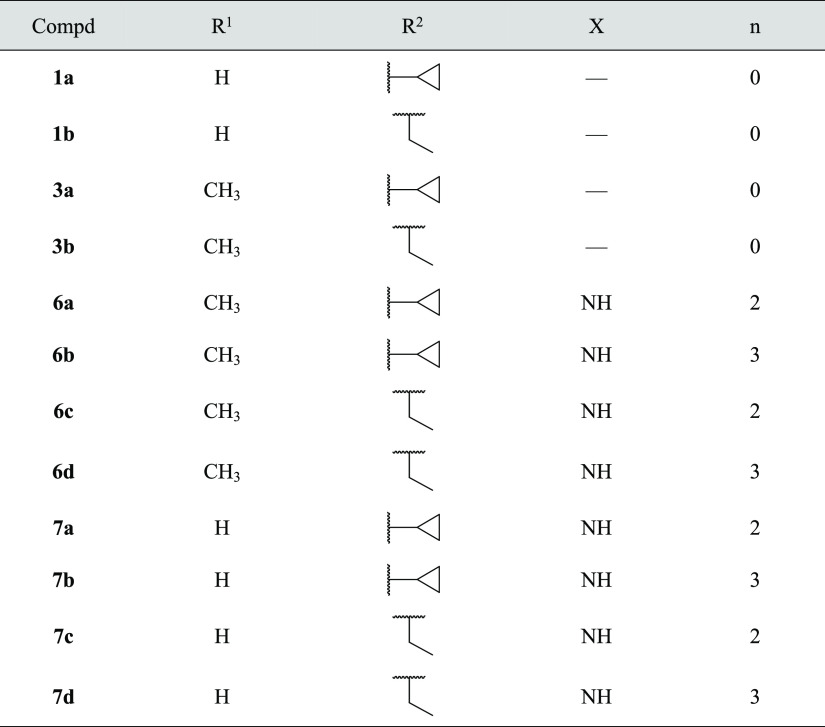
General Structure of Novel Ciprofloxacin and Norfloxacin NO Photo-Donor Hybrids

**Scheme 1:**
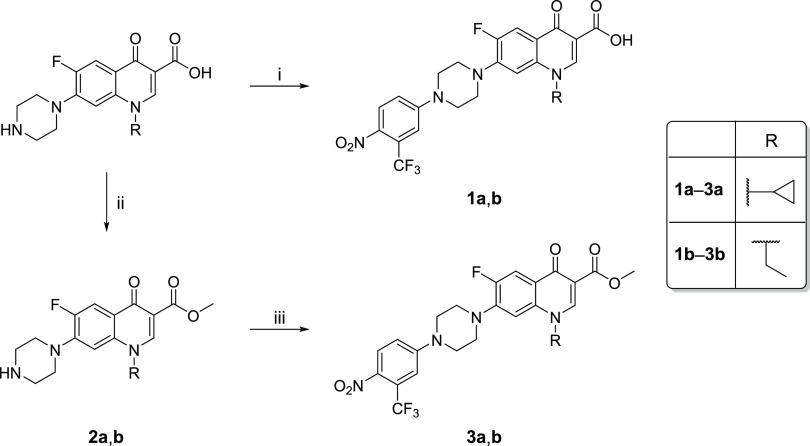
Synthetic Strategy for the Synthesis of Compounds **1**–**3a,b** Reagents and conditions: (i) 4-fluoro-1-nitro-2-(trifluoromethyl)benzene, DMSO, 120 °C, 1 h; (ii) CH_3_OH, *p*-TsOH, 70 °C, 22 h; (iii) 4-fluoro-1-nitro-2-(trifluoromethyl)benzene, CH_3_CN, 80 °C, overnight.

The strategy developed for synthesizing final compounds **1a**, **1b**, **3a**, and **3b** is depicted in Scheme 1. Carboxylic acids **1a** and **1b** were prepared following a one-step procedure involving an aromatic nucleophilic substitution between 4-fluoro-1-nitro-2-(trifluoromethyl)benzene and Cip or Nor in DMSO at 120 °C for 1 h. Methyl esters **3a** and **3b** were synthesized in two steps, including a Fisher esterification between Cip or Nor with refluxing methanol, and *p*-toluenesulfonic acid (22 h) to afford esters **2a** and **2b** that were subsequently reacted with 4-fluoro-1-nitro-2-(trifluoromethyl)benzene in refluxing acetonitrile overnight to provide final compounds **3a** and **3b**.

**Scheme 2:**
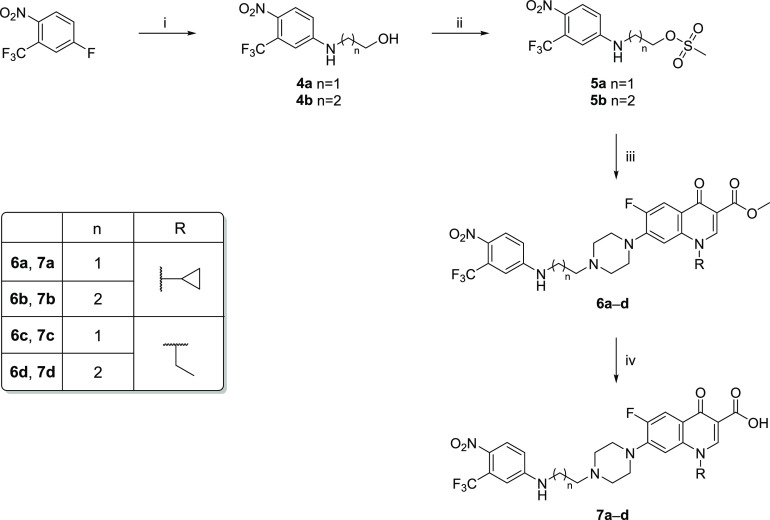
Synthetic Strategy for the Synthesis of Final Compounds **6a–6d** and **7a–7d** Reagents and conditions: (i) 1-aminoethanol or 1-aminopropan-3-ol, CH_3_CN, 60 °C, overnight; (ii) CH_3_SO_2_Cl, TEA, dry CH_2_Cl_2_, 0 °C, then room temperature, 1 h; (iii) **2a** and **2b**, CH_3_CN, reflux, overnight; (iv) aqueous NaOH 2 M, reflux, 24 h.

Scheme 2 reports the synthetic pathway to achieve final compounds **6a**–**6d** and **7a**–**7d**. Starting from 4-fluoro-1-nitro-2-(trifluoromethyl)benzene, intermediates **4a** and **4b** were prepared through an aromatic nucleophilic substitution with 2-aminoethanol or 3-aminopropan-1-ol in acetonitrile at 60 °C overnight. Mesylation of the alcoholic function of compounds **4a** and **4b** provided the intermediates **5a** and **5b** that were reacted with derivatives **2a** and **2b** in refluxing acetonitrile overnight. The obtained methyl esters **6a**–**6d** were hydrolyzed with a refluxing NaOH aqueous solution (2 M) for 24 h to afford the corresponding carboxylic acids **7a**–**7d**.

### Spectroscopic and Photochemical Characterization of Novel Ciprofloxacin and Norfloxacin Derivatives

**Figure 3:**
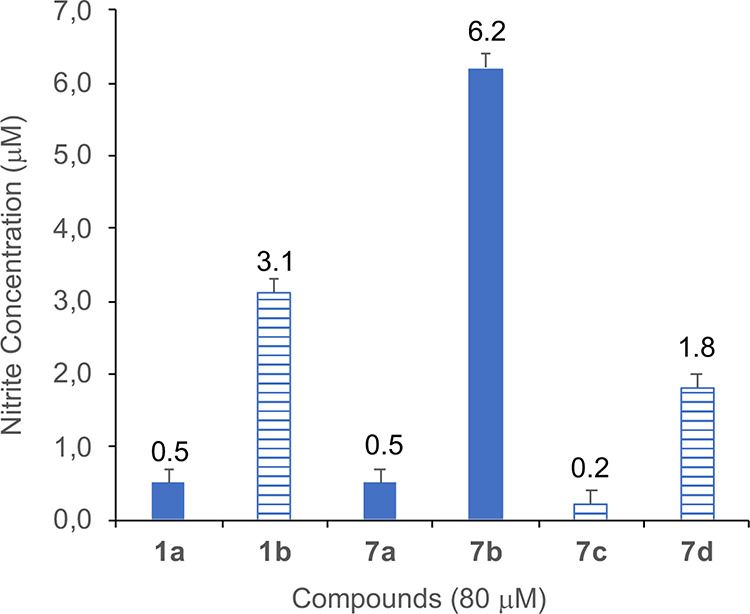
NO detection by a Griess test. Effects of 80 μM solution of **1a**, **1b**, and **7a**–**7d** on NO production after 1 h of white light irradiation (mean ± SD of three independent experiments). Nitrite concentration was determined by comparing the test samples’ absorbance values to a standard curve generated by serial dilution of 50 μM NaNO_2_.

To investigate the spectroscopic behavior of the synthesized compounds, carboxylic acids **1a**, **1b**, and **7a**–**7d** were selected for recording absorption and fluorescence spectra, showing the characteristic peaks at 400 and 450 nm, respectively (Figure S25). The amount of NO released from the selected compounds upon light irradiation was quantified using the Griess assay, in which NO_2_, generated upon the reaction of released NO with oxygen, reacts with the Griess reagent, generating a purple azo dye that can be spectroscopically monitored following its absorption peak at ∼540 nm.^43^ Therefore, aqueous solutions of compounds **1a**, **1b**, and **7a**–**7d** (80 μM) were treated with the Griess reagent and irradiated with a white lamp for different time intervals (15 min, 1 h, and 2 h). All performed analyses showed the development of a light purple coloration, confirming a weak NO release, especially after 1 h irradiation time. Although the absorption peak of the analyte solution may weakly skew absorbance measurements at 540 nm, the obtained data indicate a trend in NO production; in particular, the amount of NO released seems to be in the order **7b** > **1b** > **7d** > **1a** = **7a** > **7c**. Furthermore, the nitrite concentration was quantified by measuring the absorbance at 540 nm with respect to a standard curve of NaNO_2_ in H_2_O. Data reported in Figure 3 confirmed a significant nitrite production by **7b**, **1b**, and **7d** following 1 h irradiation, particularly evident for **7b** ([NO_2_^–^] = 6.2 μM), while the extent of NO_2_ generated by other compounds was almost negligible. Except for compound **1b**, these results suggest that longer spacers between the piperazine ring and the NO-donor moiety might favor the NO production yield.

### *In Vitro* Antitumor Activity Evaluation of Novel Fluoroquinolone Derivatives

**Figure 4:**
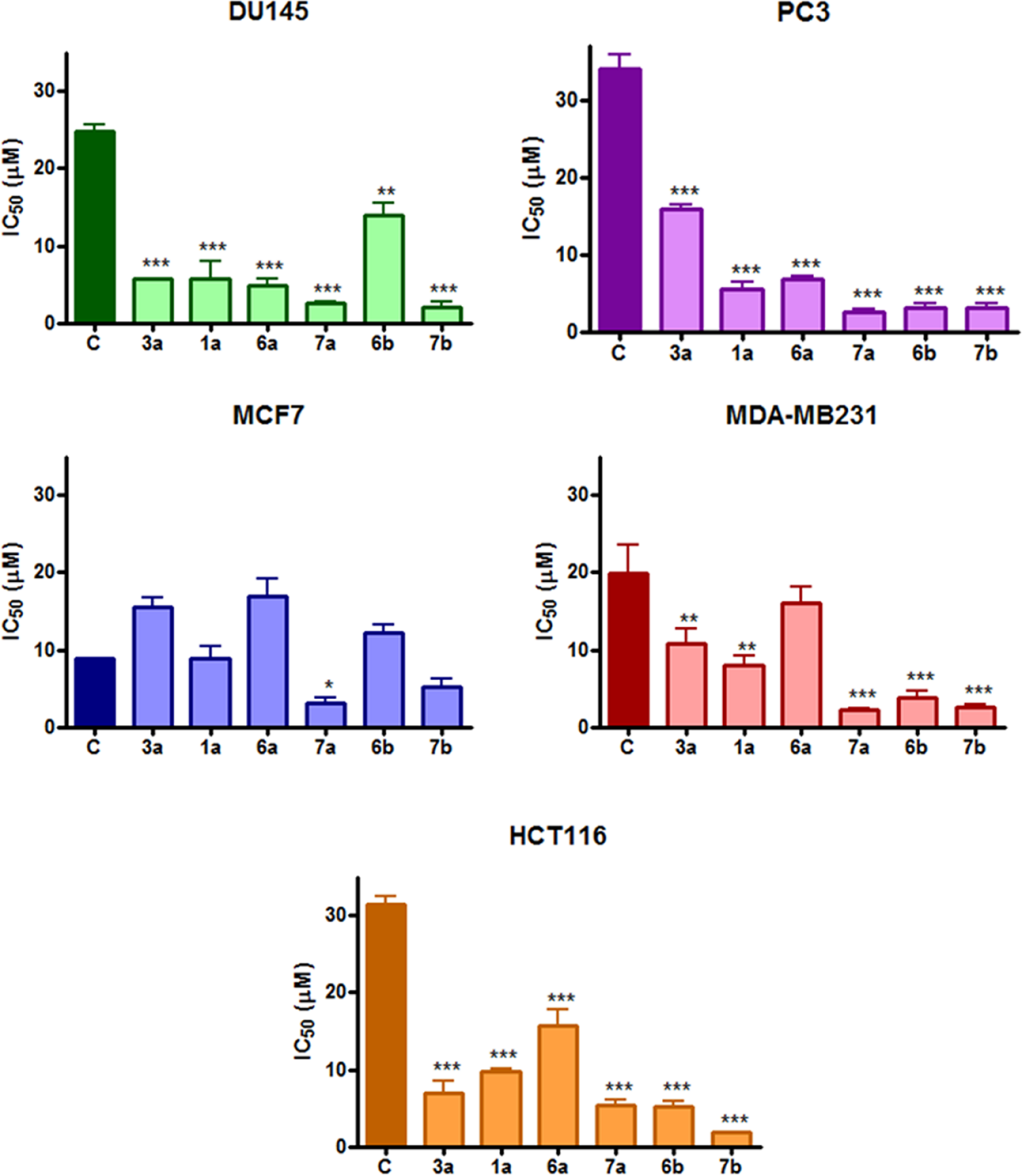
IC_50_ values obtained following 72 h treatment with Ciprofloxacin (C) and its derivatives in the MTT assay (mean ± ES 4/5 independent experiments; *p < 0.05, **p < 0.01, ***p < 0.001 *vs* Cip).

**Figure 5:**
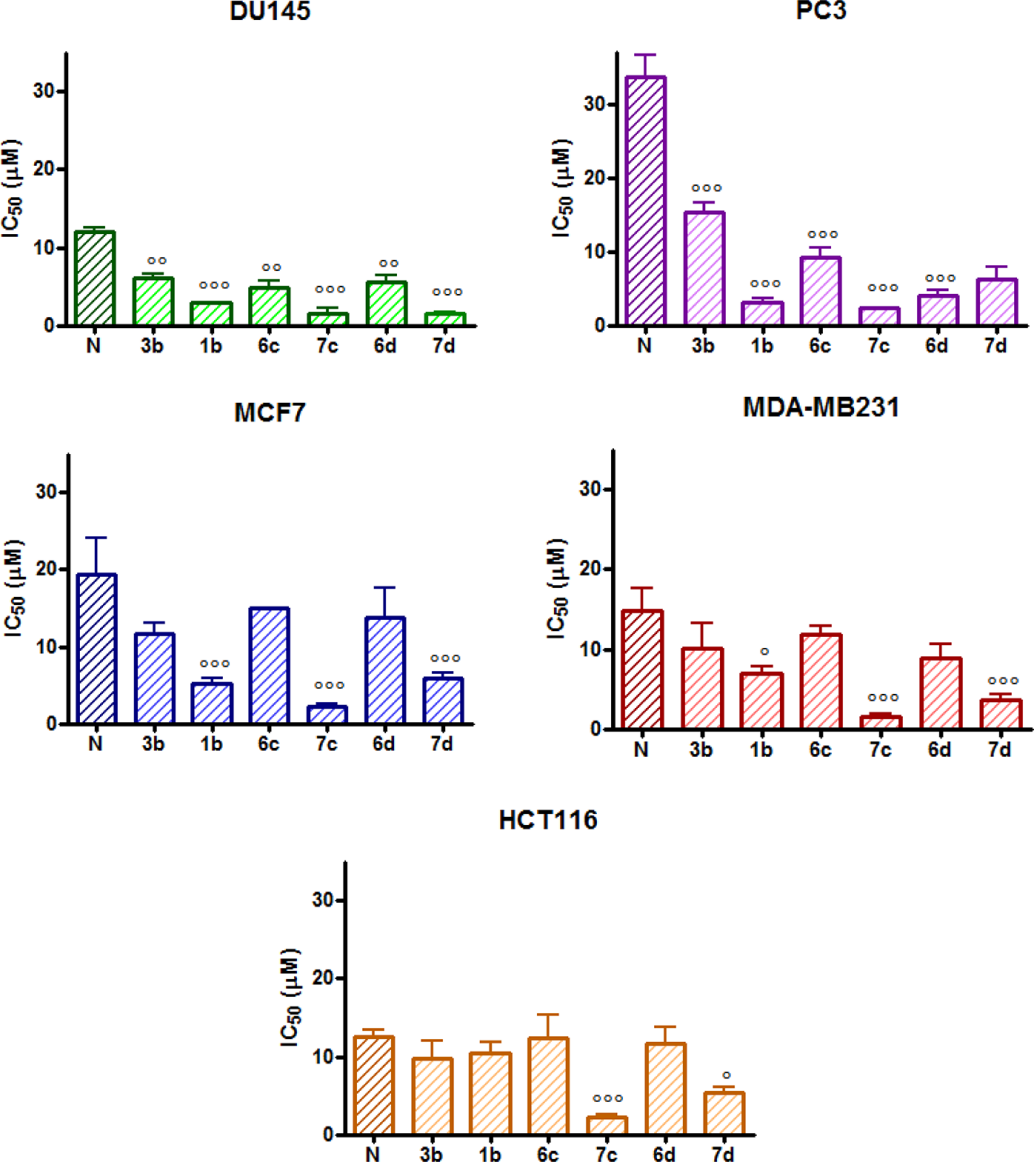
IC_50_ values obtained following 72 h treatment with Norfloxacin (N) and its derivatives in the MTT assay (mean ± ES 4/5 independent experiments; °*p* < 0.05, °°*p* < 0.01, °°°*p* < 0.001 *vs* Nor).

The effect of the novel hybrid derivatives on cellular viability of a panel of tumor cell lines of different tissue origins (DU145 and PC3: prostate; MCF-7, MCF7/ADR, and MDA-MB231: breast; HCT116: colon) was investigated through the MTT assay following 3 days treatment with the compounds. Histograms reported in Figures 4 and 5 represent the IC_50_ values extrapolated from the corresponding concentration–response curves. In each graph, IC_50_ values of new derivatives were compared to the reference compounds, *e.g.*, Cip or Nor. All compounds showed effects at micromolar concentrations (Tables S1 and S2).

Figure 4 shows that all Cip derivatives were significantly more potent than the parent compound in DU145 and PC3 prostate cancer cells in HCT116 colorectal cancer cells (the more potent being compound **7b**, with an IC_50_ = 1.83 μM) and in MDA-MB231 breast cancer cells (IC_50_ values lower than 2.50 μM for compounds **7a** and **7b**) while only compounds **7a** and **7b** were more potent than Cip in MCF7 cells (Table S1). In general, carboxylic acids **7a** and **7b**, where the alkyl linker connects the fluoroquinolone scaffold to the NO photo-donor moiety, showed lower IC_50_ values when compared to their corresponding methyl esters **6a** and **6b**. Specifically, this trend was more pronounced for compound **7a**, which was 2- to 7-fold more potent than **6a** in the tested cell lines. In contrast, the difference in potency was lower when comparing **6b** and **7b**. These data indicate that a shorter alkyl bridge improves the cytotoxic effect (**7a***vs***7b**). On the other hand, results obtained for methyl esters **6a** and **6b** suggest that a longer alkyl chain provides better cytotoxic properties. Finally, compounds lacking the alkyl bridge (**1a** and **3a**) displayed an ambiguous trend on the tested cell lines. Indeed, DU145 and HCT116 cell lines were more sensitive to the methyl ester **3a**, whereas the PC3 and MDA-MB231 cell lines displayed a slight susceptibility toward the carboxylic acid **1a**.

A similar trend was observed for the SAR of the novel Nor hybrids, except for the better activity of carboxylic acid **1b** in all the tested cell lines when compared to its methyl ester analog **3b**. In particular, results obtained in DU145 and PC3 cells showed that all Nor derivatives were significantly more potent than the reference compound, with best results obtained for **7d** in the DU145 cell line (IC_50_ = 1.56 μM) and **7c** in the PC3 cell line (IC_50_ = 2.33 μM). In MCF7 and MDA-MB231 cells, compounds **1b** and **7d** showed a significantly lower IC_50_ than Nor, while **7c** was the most potent among all the novel hybrids in both breast cancer cell lines (IC_50_ = 2.27 and 1.52 μM for MCF7 and MDA-MB231, respectively). Interestingly, in HCT116 cells, only **7c** and **7d** resulted to be more potent than Nor (Figure 5 and Table S2).

Last, the effects of all the newly synthesized compounds on cell viability were not limited to cancer cell lines; indeed, as shown on Table 2, which report the IC_50_ values obtained by the MTT assay following 72 h treatment with the compounds, also the non-tumorigenic HBL100 and the WH1 fibroblast cell lines responded to Cip, Nor, and to their derivatives at similar extent compared to cancer cell lines (Table 2).

**Table 2:** IC_50_ Values Obtained with the MTT Assay following 72 h Treatment with Ciprofloxacin (Cip), Norfloxacin (Nor), and Their Derivatives

	IC_50_ (μM) ± ES^a^			IC_50_ (μM) ± ES^a^	
Compd	WH1	HBL100	Compd	WH1	HBL100
**1a**	9.17 ± 0.60	7.79 ± 0.66	**1b**	5.35 ± 1.14^b^	9.26 ± 1.79
**3a**	8.38 ± 1.20	10.57 ± 2.40	**3b**	9.00 ± 1.91	11.95 ± 1.70
**6a**	10.5 ± 2.50	9.76 ± 0.90	**6c**	10.63 ± 1.90	10.85 ± 0.60
**6b**	13.2 ± 3.40	12.21 ± 2.00	**6d**	9.67 ± 1.65	3.68 ± 0.20^b^
**7a**	5.70 ± 0.92	9.14 ± 0.44	**7c**	3.51 ± 0.40^b^	11.09 ± 0.20
**7b**	7.05 ± 1.20	9.19 ± 0.66	**7d**	8.94 ± 0.91	10.40 ± 0.90
Cip	14.2 ± 2.10	10.28 ± 1.00	Nor	14.07 ± 2.10	12.43 ± 0.80

a Mean ± ES 4/5 independent experiments.

b *p* < 0.05 *vs* Nor.

**Figure 6:**
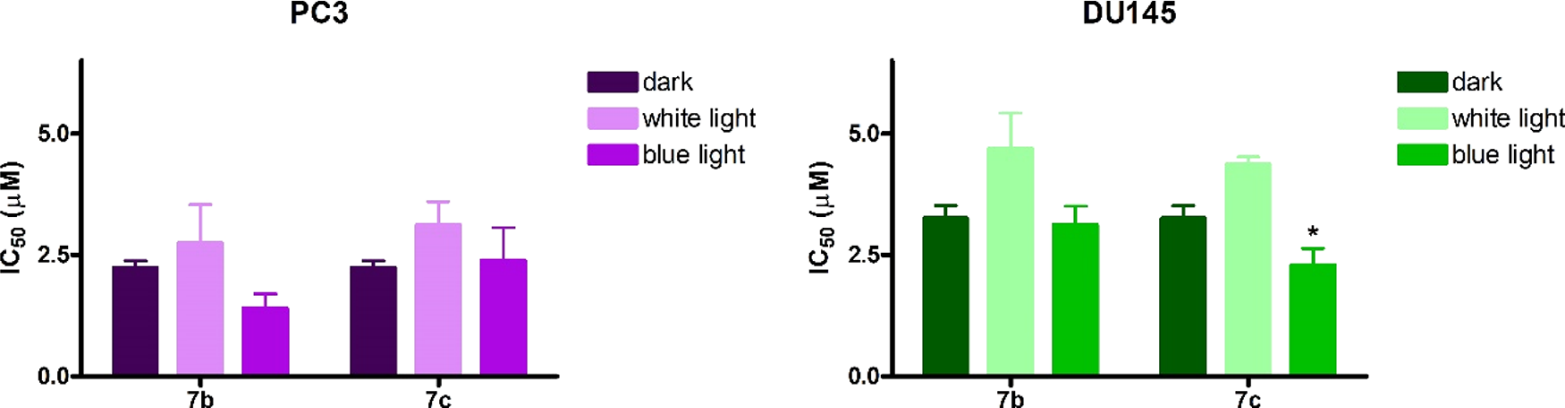
IC_50_ values obtained following 24 h treatment with compound **7b** or **7c** with 1 h irradiation and 48 h incubation in the drug-free medium and MTT assay (mean ± ES 3/4 independent experiments. *p < 0.05 *vs* white light).

To evaluate if NO released upon light irradiation could affect cell viability, the MTT assay was performed on DU145 and PC3 cells following treatment with two compounds that showed interesting potency against the two prostate cancer cell lines and different degrees of NO release. Specifically, the Cip and the Nor derivative that showed to develop the highest and the lowest NO levels by the Griess assay, namely, **7b** and **7c**, respectively, were selected for this experiment. Treatment was followed by 1 h irradiation with white or blue light and 48 h incubation in a drug-free medium. As shown in Figure 6, no differences in IC_50_ values were observed in cells treated with **7b** and **7c** and irradiated (both with white or blue light) with respect to cells treated and kept in the dark. However, compound **7c** seems to be more active on DU145 cells when irradiated under blue light with respect to white light.

Overall, these results might indicate that the amount of NO released could be inadequate to induce cell death, thus confirming that the NO release does not play an additive role in the new fluoroquinolone derivatives’ toxic effect.

A significant obstacle to the successful chemotherapeutic treatment of tumors is their inadequate response to anticancer drugs due to intrinsic or acquired resistance phenomena. Tumor cells can be insensitive to drug treatment at the therapy onset (intrinsic resistance), or they can initially respond to anticancer agents, becoming refractory on subsequent treatment cycles (acquired resistance). For instance, the development of MDR following an initial drug response severely limits the success of doxorubicin (DOX) treatment of breast cancers.^44,45^ DOX is an anthracycline antibiotic targeting topoisomerase II and represents a mainstay in clinical management of early-stage and metastatic breast cancer. MDR to DOX involves few biochemical alterations, such as reduced drug accumulation, increased detoxification, increased DNA repair, topoisomerase II alterations, or in cell cycle regulation. In this scenario, identifying MDR-modulating agents or drugs able to escape MDR phenomena is of utmost importance in anticancer research.

**Figure 7:**
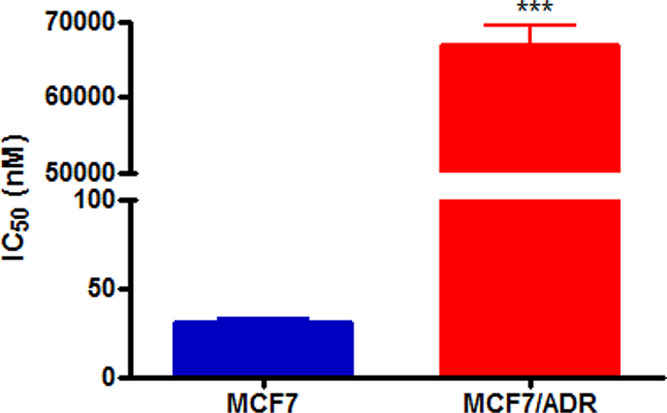
IC_50_ values obtained following 72 h treatment with DOX and the MTT assay (mean ± ES 4/5 independent experiments; ***p < 0.001 *vs* MCF7).

As expected, the DOX-resistant MCF7/ADR cell line, obtained by selecting MCF7 cells exposed to increasing DOX concentrations, is significantly less sensitive to anthracycline treatment with respect to the wild-type cell line, with a resistance index (R.I.) of 22 (Figure 7). Interestingly, MCF7/ADR cells showed similar sensitivity to several Cip and Nor derivatives (Table 3) as observed for the MCF7 cell line, with the best IC_50_ values obtained for carboxylic acids **1a** and **1b** (8.72 and 5.63 μM, respectively).

**Table 3:** IC_50_ Values Obtained with the MTT Assay following 72 h Treatment with DOX and Ciprofloxacin (Cip)/Norfloxacin (Nor) Derivatives

	IC_50_ (μM) ± ES^a^				IC_50_ (μM) ± ES^a^		
Compd	MCF7	MCF7/ADR	R.I.	Compd	MCF7	MCF7/ADR	R.I.
**1a**	8.80 ± 1.59	8.72 ± 1.25	1.0	**1b**	5.24*^b^* ± 0.70	5.63*^b^* ± 0.07	1.1
**3a**	15.45 ± 1.20	19.55 ± 3.20	1.3	**3b**	11.68 ± 1.40	15.04 ± 2.10	1.3
**6a**	16.84 ± 2.40	15.12 ± 2.30	0.9	**6c**	15.04 ± 0.10	17.15 ± 1.00	1.1
**6b**	12.12 ± 1.10	18.76 ± 0.10	1.5	**6d**	10.53 ± 1.20	28.04 ± 1.10	2.7
**7a**	3.12 ± 0.62	14.69 ± 4.20	4.7	**7c**	2.27*^b^* ± 0.31	18.31 ± 1.40	8.1
**7b**	5.23 ± 0.82	23.55 ± 3.60	4.5	**7d**	5.83 ± 0.82	20.02 ± 0.30	3.4
Cip	8.85 ± 0.09	19.12 ± 0.50	2.2	Nor	19.37 ± 0.70	20.06 ± 1.40	1.0
DOX	0.031 ± 0.001	66.90 ± 2.56	22				

a Mean ± ES 4/5 independent experiments.

b *p* < 0.05 *vs* reference compound.

**Figure 8:**
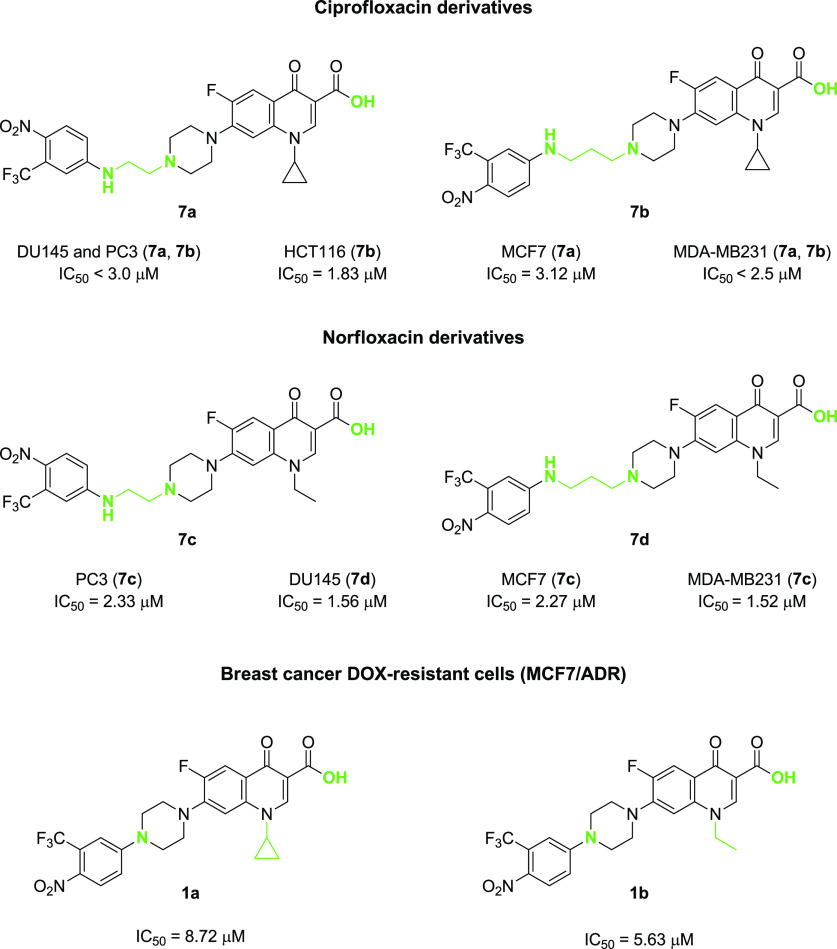
Summary of the most potent compounds synthesized in this work with their IC_50_ values on the tested cancer cell lines.

In summary, the *in vitro* experiments showed that derivatives **7a** and **7b** are the most effective Cip analogs against all considered cell lines and with respect to the reference compound, indicating that regardless of the linker length, the carboxylic acid moiety outperforms the ester function (Figure 8). In line with these results, Nor derivatives **7c** and **7d** displayed a similar trend, and compound **7c** was the best performing of the series (Figure 8). Cytotoxicity studies performed on MCF7/ADR resistant cell lines confirmed that the carboxylic moiety is essential for the anticancer activity and that only derivatives **1a** and **1b**, where the *p*-nitro-trifluoromethyl aniline is directly linked to the fluoroquinolone scaffold, can provide IC_50_ values in the low micromolar range (Figure 8).

### *In Vitro* Antimicrobial Activity on *Pseudomonas aeruginosa*

Considering that fluoroquinolones, such as Cip and Nor, are among the few antibiotics used to control the growth of *P. aeruginosa*, an opportunistic pathogen representing one of the most relevant agents of nosocomial infections, we preliminarily determined whether the novel derivatives could also be effective against the model strain PAO1. Our results indicate that, while Cip and Nor had minimal inhibitory concentration (MIC) values of 1.82 ± 1.19 and 4.16 ± 1.80 μg/mL, respectively, none of our newly designed derivatives showed antimicrobial activity comparable or better than reference compounds (MIC ∼25 μg/mL). These results might indicate that the fluoroquinolone scaffold’s structural changes compromise the antimicrobial activity, probably due to an impaired interaction with the microbial cell wall and/or with the bacterial target, *e.g.*, the DNA gyrase. To rule out the first hypothesis, we investigated the ability of our novel compounds to bind the PAO1 cell wall. Binding experiments showed that upon incubation of *P. aeruginosa* PAO1 cells with our fluoroquinolones, a decrease in absorbance was observed in the supernatants (Figures S26 and S27), indicating good binding rates with the outer layer of the cell wall. However, it should be underlined that a good interaction between the molecules and the bacterial cell wall is not predictive of an effective uptake.

**Figure 9:**
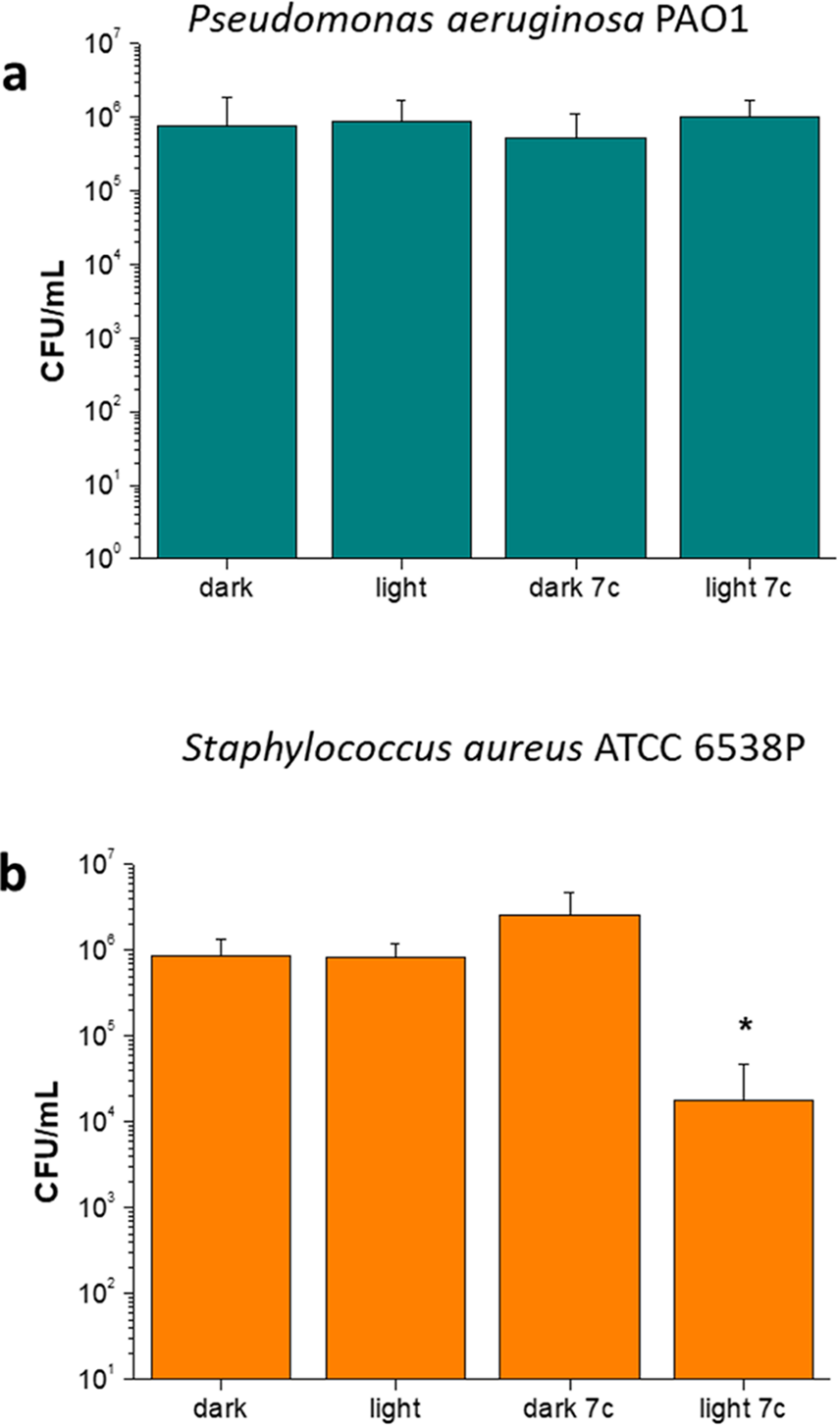
Photodynamic treatment of *P. aeruginosa* PAO1 (a) and *S. aureus* ATCC 6538P (b). Bacteria were incubated in the dark with or without **7c** (10 μM) for 10 min and then irradiated under blue light (20 J/cm^2^). Dark controls were not irradiated. Viable cells are expressed as CFU/mL. Values represent the mean of at least three independent experiments, *p < 0.05.

Since most compounds showed an absorption peak in the 390–395 nm region, we next investigated their activity upon irradiation with blue light. For a preliminary evaluation, we selected compound **7c**, showing the highest absorption peak in the violet-blue range and some degree of activity against DU145 cancer cells. Upon irradiation with a light-emitting diode at 405 ± 10 nm and a fluence not toxic to cells (20 J/cm^2^), compound **7c** did not show any antimicrobial activity against *P. aeruginosa* PAO1 (Figure 9a), indicating that despite its interaction with the cell wall, this is not sufficient to induce killing by photo-oxidative stress.

It is well established that one of the major challenges in treating Gram-negative bacteria, with respect to the Gram-positive ones, is the difficulty of antimicrobial agents to strongly bind and cross their cell wall, which significantly differs in composition as compared to the latter. In order to shed light on the specific behavior of our fluoroquinolones, we then evaluated the effectiveness of compound **7c** in killing Gram-positive *Staphylococcus aureus*. Interestingly, the photoactivation of **7c** caused a significant (*p* < 0.05) 2 log unit decrease in *S. aureus* with respect to the dark incubated control (Figure 9b). This observation might support the hypothesis that compound **7c** is able to efficiently bind the cell wall and probably cross the cytoplasmic membrane of *S. aureus*, ultimately inducing a photo-oxidative stress at the cell-wall level and/or at the cytoplasmic level, thus causing cellular death upon light irradiation. This result indirectly further demonstrates that despite the fact that our fluoroquinolones bind the cell wall of *P. aeruginosa*, this is not sufficient to induce bacterial killing by photoactivation. The selective activity of derivative **7c** toward Gram-positive bacteria indicates that our derivatives act as neutral antimicrobial photosensitizers (aPSs). In fact, it has been reported that neutral photosensitizers are usually active toward Gram-positive microorganisms and not in the Gram-negative ones, being able to overcome the murein barrier of the first, but not the outer membrane of the cell wall of the latter.^46^ Based on the observed selectivity of **7c**, it could be hypothesized that despite the fact that molecular modeling studies (see the following paragraph) account for the effective binding of fluoroquinolones with both topoisomerase II and gyrase, their biological activity against *P. aeruginosa* is hampered by an ineffective uptake. In the future, this could be overcome by incorporating these molecules within suitably designed carriers.

### Computational Studies

Molecular modeling studies were carried out to investigate the interactions with the reference cellular and bacterial targets. The calculated free energies of binding (Δ*G*) to the catalytic site of the human topoisomerase IIα (Topo IIα), bacterial topoisomerase IIA (Topo IIA), and bacterial gyrase (Gyr) for the novel compounds are reported in Table 4. First, the crystal structure of the Topo IIα isoform (PDB ID: 5GWK) was used for docking studies.^47^

**Table 4:** Calculated Free Energies of Binding (Δ*G*, in kcal/mol) of the Novel Compounds to the Catalytic Sites of Topo IIα, Topo IIA, and Gyr

Compd	calcd. Δ*G* human Topo IIα	calcd. Δ*G* bacterial Topo IIA	calcd. Δ*G* bacterial Gyr
Cip	–9.1	–11.1	–7.9
Nor	–8.5	–10.6	–7.0
**1a**	–11.6	–11.8	–8.4
**1b**	–10.8	–10.8	–8.1
**3a**	–10.8	–9.68	–8.1
**3b**	–10.7	–12.1	–9.3
**6a**	–10.7	–13.8	–9.8
**6b**	–10.1	–10.7	–8.1
**6c**	–10.5	–11.9	–9.2
**6d**	–10.0	–13.4	–9.4
**7a**	–11.3	–13.8	–9.7
**7b**	–11.4	–14.1	–8.9
**7c**	–10.6	–12.9	–9.2
**7d**	–13.2	–15.1	–10.5
DOX	–13.5	–12.5	–12.5

**Figure 10:**
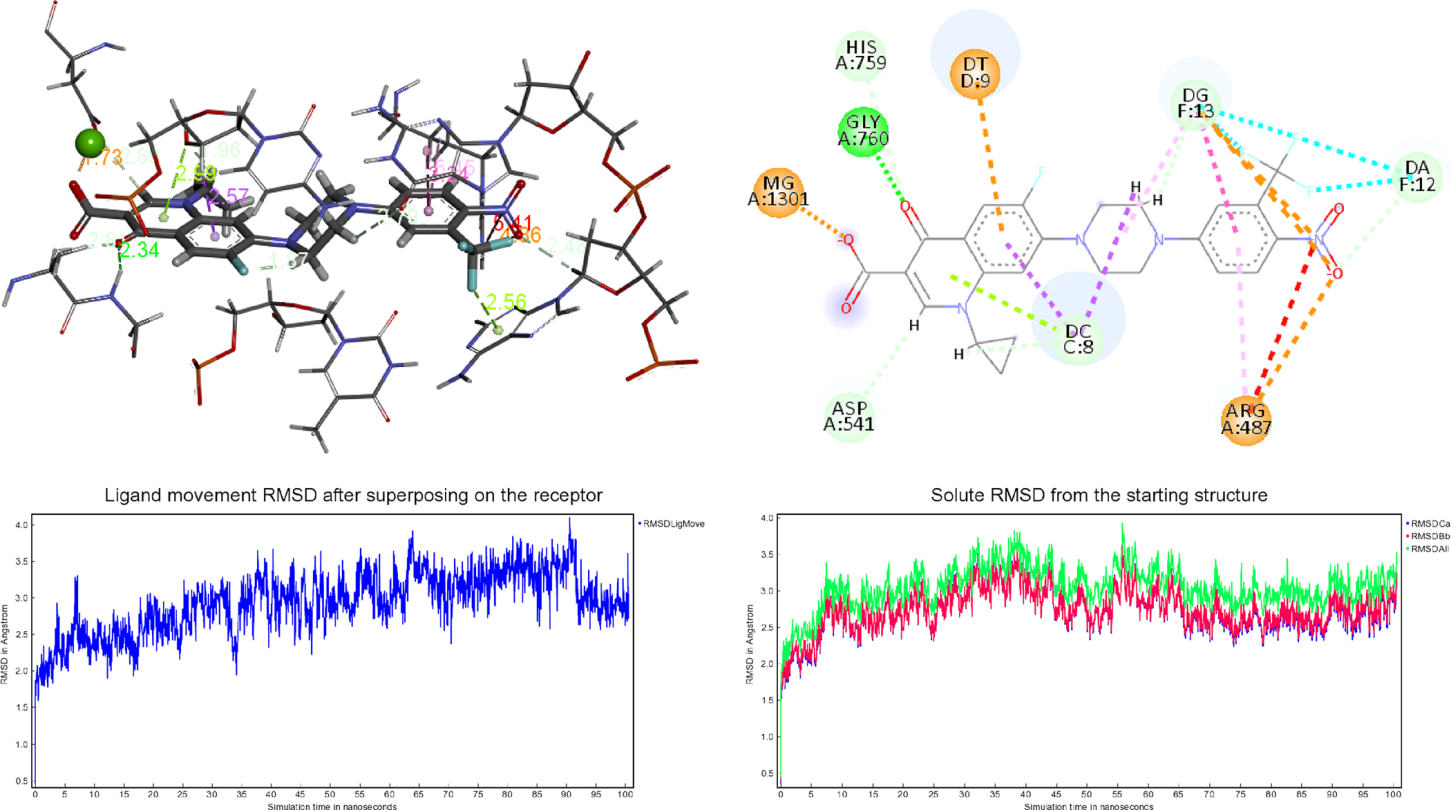
3D superposition of the best-docked pose for **1a** bound to the Topo IIα (A) and binding site interactions (B). RMSD superposing on the receptor (C) and starting structure (D).

All compounds displayed better *in silico* affinity toward Topo IIα than Cip and Nor, while DOX showed greater affinity, as demonstrated by experimental data. The Cip derivatives have lower free energies of binding than the Nor derivatives, except for compounds **6d** and **7d**. The **1a** carboxyl group coordinates Mg^2+^ via a salt bond of 1.73 Å (Figure 10A). Furthermore, the complex is stabilized, within the catalytic site, by the H-bond with the Gly760 and Asp541 backbone, while the nitro group establishes electrostatic interactions with Arg487 (Figure 10B).

The protein aids the anchoring of the ligand within the pocket by a cation-π link formed between Arg487 and the nitro group portion of the ligand. The neighboring DNA bases also contribute to the stabilization of the complex.

Once the general interaction model was established, equilibrium molecular dynamics (MD) simulations (100 ns) were performed to analyze the Topo IIα/DNA/**1a** ternary model system’s evolution and stability. The RMSDs of the tertiary structures (Topo IIα/DNA/**1a**) compared to the first ones at time 0 were analyzed and plotted during the 100 ns MD simulation (Figure 10C). The overall RMSD for the protein system appeared to have reached the equilibrium after 10 ns (Figure 10D), and the stabilization of the protein–ligand complex was reached after 7 ns, keeping the complex’s extensive hydrogen-bonding network constant. The energies of binding, calculated by the Molecular Mechanics Poisson–Boltzmann Surface Area (MM/PBSA) methodology (see the Experimental Section), including the time average, along MD simulation trajectories were employed to assess the strength of the interactions between the ligand and the binding pocket in the dynamic environment. Compound **1a** shows a stable fluctuation that settles after the first 10 ns and records an average value of −17.4 kcal/mol in the remaining 90 ns (Figure S28).

The docked laying of **1b** is very similar to that of **1a** despite a loss of 0.8 kcal/mol, probably due to the ethyl group’s worse stacking. It is interesting to note that compounds **7a**, **7b**, and **7d** have lower calculated free energies of binding than other compounds against human Topo IIα. Indeed, these compounds have shown excellent results in the tested cancer cell lines.

All the novel compounds were anchored in the active sites of Topo IIA (PDB ID: 2XCT) of *S. aureus* and DNA gyrase B (PBD ID: 6MS1) of *P. aeruginosa*. Anchored poses with the lowest bond energy, hydrogen bonds, noncovalent interactions, such as the π–π interactions, and the details of the π-cationic interactions were recorded and validated.

All the compounds analyzed showed better *in silico* affinity on bacterial topoisomerase than Cip and Nor, except compound **3a**, while all compounds showed lower *in silico* activity against DNA gyrase. Despite the good *in silico* results, the compounds have a worse antimicrobial activity *in vitro* than the reference drugs (Cip and Nor). It seems that the novel compounds bind to the outer membrane of the Gram-negative cell wall, but the crossing of the cytoplasmic membrane to reach the cytoplasmic environment and then the molecular target could represent the issue to overcome. Thus, further investigations are needed to shed light on the observed impairment of novel derivatives’ antimicrobial activity.

## Conclusions

In this work, we successfully managed to design and synthesize twelve novel Cip and Nor derivatives endowed with a NO photo-donor moiety. The light-triggered release of NO has been demonstrated by spectroscopic and photochemical studies, showing the release of this gasotransmitter in the micromolar range, especially for compound **7b**. Docking studies confirmed that these novel chemical entities effectively bind to both bacterial and human topoisomerases, with better calculated free binding energies with respect to the parent compounds. *P. aeruginosa* PAO1 was not sensitive to the novel derivatives, and photoactivation experiments support the hypothesis that this could be ascribable to an inefficient uptake.

As far as anticancer activity is concerned, all novel fluoroquinolone derivatives displayed strong anticancer potency on a panel of different cancer cell lines, which was especially remarkable for compounds **7a**–**7d**. On the other hand, the light-triggered release of NO from compounds **7b** and **7c** did not grant an additional cytotoxic effect on PC3 and DU145 prostate cancer cell lines, although a better response to the compounds was observed following blue light irradiation with respect to white light irradiation. Further studies focused on the precise mechanism of action of these compounds and on the role of NO in these cell lines are in progress.

Importantly, our data showed that some of the tested compounds, including compounds **1a**, **1b**, and **7a**–**7d**, exhibit cytotoxic effects on MDA-MB231 cells, which are representative of triple-negative breast cancer (TNBC), one of the most aggressive and refractory forms of breast cancer. TBNC does not respond to endocrine therapy or other currently available targeted agents;^48−50^ thus, alternative therapeutic options that can selectively address this tumor subset are urgently needed. In this view, the promising results obtained with our novel derivatives on MDA-MB231 cells represent an encouraging starting point for developing and optimizing more effective treatment. Furthermore, compounds **1a** and **1b** also displayed a strong cytotoxic effect on DOX-resistant MCF7/ADR breast cancer cells, making these hybrids promising candidates for MDR breast cancer treatment. As expected, most of our fluoroquinolone derivatives displayed a certain extent of toxicity on healthy cells; this issue could be overcome in the future by encapsulating those molecules within suitably designed delivery systems.

## Experimental Section

### General Remarks

Reagent-grade chemicals were purchased from Sigma-Aldrich or Fluorochem and were used without further purification. All reactions were monitored by thin-layer chromatography (TLC) performed on silica gel Merck 60 F254 plates; the spots were visualized by UV light (λ = 254 and 366 nm) and an iodine chamber. Melting points were determined on a Büchi B-450 apparatus in capillary glass tubes and are uncorrected. Flash chromatography purification was performed on a Merck silica gel 60, 0.040–0.063 mm (230–400 mesh) stationary phase using glass columns with a diameter between 1 and 4 cm. Nuclear magnetic resonance spectra (^1^H NMR and ^13^C NMR recorded at 500 and 125 MHz, respectively) were obtained on Varian INOVA spectrometers using CDCl_3_, acetone-*d*_6_, CD_3_OD, and DMSO-*d*_6_ with a 0.03% of TMS as an internal standard. Coupling constants (*J*) are reported in hertz. Signal multiplicities are characterized as s (singlet), d (doublet), t (triplet), q (quartet), m (multiplet), br (broad), and app (apparent). Purities of all compounds were ≥95% as determined by microanalysis (C, H, and N) that was performed on a Carlo Erba instrument model E1110; all the results agreed within ±0.4% of the theoretical values.

### General Procedure for the Synthesis of Carboxylic Acids **1a** and **1b**

To a suspension of Cip or Nor (0.100 g, 0.3 mmol) in 5 mL of DMSO was added 4-fluoro-1-nitro-2-(trifluoromethyl)benzene (0.063 g, 0.3 mmol) under stirring. The color of the suspension immediately turned yellow. The reaction was carried out in a sealed Pyrex vial at 120 °C for 60 min. After cooling to room temperature, the product was precipitated with deionized water and decanted. The solid yellow residue was repeatedly washed in sequence with deionized water, isopropanol, and diethyl ether. The obtained solid was dried under N_2_ and did not require any further purification. According to this procedure, the following products have been obtained.

#### 1-Cyclopropyl-6-fluoro-7-(4-(4-nitro-3-(trifluoromethyl)phenyl)piperazin-1-yl)-4-oxo-1,4-dihydroquinoline-3-carboxylic Acid (**1a**)

Yellow solid (89%): mp 296–298 °C; ^1^H NMR (500 MHz, DMSO-*d*_6_): δ 8.67 (s, 1H), 8.13 (d, *J* = 9.5 Hz, 1H), 7.94 (d, *J* = 13.5 Hz, 1H), 7.59 (d, *J* = 7.5 Hz, 1H), 7.37 (d, *J* = 2.5 Hz, 1H), 7.31 (dd, *J* = 9.5, 2.5 Hz, 1H), 3.86–3.82 (m, 1H), 3.78 (t, *J* = 5.0 Hz, 4H), 3.54 (t, *J* = 4.8 Hz, 4H), 1.32 (q, *J* = 7.0 Hz, 2H), 1.22–1.19 (m, 2H); ^13^C NMR (125 MHz, DMSO-*d*_6_): δ 176.35, 165.92, 152.89, 148.10, 144.59 (d, *J*_CF_ = 9.9 Hz), 139.19, 135.59, 128.85, 124.33, 121.36, 118.59, 115.29, 111.67 (d, *J*_CF_ = 5.1 Hz), 111.08 (d, *J*_CF_ = 22.3 Hz), 106.76, 106.18, 48.53, 45.96, 35.87, 7.60. Anal. Calcd. for C_24_H_20_F_4_N_4_O_5_: C, 55.39; H, 3.87; N, 10.77. Found: C, 55.42; H, 3.89; N, 10.74.

#### 1-Ethyl-6-fluoro-7-(4-(4-nitro-3-(trifluoromethyl)phenyl)piperazin-1-yl)-4-oxo-1,4-dihydroquinoline-3-carboxylic Acid (**1b**)

Yellow solid (74%): mp 310–312 °C; ^1^H NMR (500 MHz, DMSO-*d*_6_): δ 8.97 (s, 1H), 8.13 (d, *J* = 9.0 Hz, 1H), 7.97 (d, *J* = 13.0 Hz, 1H), 7.36 (s, 1H), 7.31 (d, *J* = 9.0 Hz, 1H), 7.22 (d, *J* = 7.0 Hz, 1H), 4.61 (q, *J* = 7.0 Hz, 2H), 3.77 (br s, 4H), 3.53 (br s, 4H), 1.43 (t, *J* = 7.0 Hz, 3H); ^13^C NMR (125 MHz, DMSO-*d*_6_): δ 176.25, 166.06, 153.06, 148.58, 144.86, 137.19, 135.77, 128.80, 124.35, 119.81, 119.32, 117.47, 115.32, 111.68 (d, *J*_CF_ = 6.25 Hz), 111.42 (d, *J*_CF_ = 28.7 Hz), 105.78, 48.64, 46.06, 41.00, 14.29. Anal. Calcd. for C_23_H_20_F_4_N_4_O_5_: C, 54.33; H, 3.96; N, 11.02. Found: C, 54.50; H, 3.94; N, 10.99.

### General Procedure for the Synthesis of Ciprofloxacin and Norfloxacin Methyl Esters **2a** and **2b**

To a warmed suspension of the starting fluoroquinolone (1.00 g, 3.00 mmol) in 100 mL of CH_3_OH was dropped *p*-toluenesulfonic acid (3.00 g, 15.7 mmol) dissolved in 5 mL of CH_3_OH via a syringe. The resulting solution was refluxed for 22 h. The reaction mixture was then cooled to room temperature, and the reaction solvent was removed under vacuum. To the resulting yellow oil was added a saturated solution of Na_2_CO_3_ (50 mL), and the aqueous phase was extracted with CH_2_Cl_2_ (3 × 50 mL). The organic phase was dried over anhydrous Na_2_SO_4_, filtered, and concentrated at reduced pressure. The crude was purified by flash chromatography using a CH_2_Cl_2_/CH_3_OH gradient eluting system. According to this procedure, the following products have been obtained.

#### Methyl 1-Cyclopropyl-6-fluoro-4-oxo-7-(piperazin-1-yl)-1,4-dihydroquinoline-3-carboxylate (**2a**)

White solid (91%): mp 230–233 °C; ^1^H NMR (500 MHz, CDCl_3_): δ 8.54 (s, 1H), 8.02 (d, *J* = 13.3 Hz, 1H), 7.29–7.27 (m, 1H), 3.91 (s, 3H), 3.46–3.42 (m, 1H), 3.29–3.27 (m, 4H), 3.15–3.13 (m, 4H), 2.40 (s, 2H), 1.33 (t, *J* = 6.5 Hz, 2H), 1.14 (q, *J* = 6.5 Hz, 2H); ^13^C NMR (125 MHz, CDCl_3_): δ 173.24, 166.61, 153.61 (d, *J*_CF_ = 247.5 Hz), 148.46, 145.15 (d, *J*_CF_ = 10.0 Hz), 138.18, 123.14 (d, *J*_CF_ = 7.5 Hz), 113.46 (d, *J*_CF_ = 22.5 Hz), 110.21, 104.87, 52.20, 51.19, 46.06, 34.65, 8.28. Anal. Calcd for C_18_H_20_FN_3_O_3_: C, 62.60; H, 5.84; N, 12.17. Found: C, 62.78; H, 5.86; N, 12.19.

#### Methyl 1-Ethyl-6-fluoro-4-oxo-7-(piperazin-1-yl)-1,4-dihydroquinoline-3-carboxylate (**2b**)

White solid (92%): mp 189–190 °C; ^1^H NMR (500 MHz, CDCl_3_): δ 8.42 (s, 1H), 8.06 (d, *J* = 13.0 Hz, 1H), 6.73 (d, *J* = 7.0 Hz, 1H), 4.20 (q, *J* = 7.5 Hz, 2H), 3.92 (s, 3H), 3.22–3.21 (m, 4H), 3.10–3.08 (m, 4H), 1.92 (s, 2H), 1.54 (t, *J* = 7.5 Hz, 3H); ^13^C NMR (125 MHz, CDCl_3_): δ 173.15, 166.72, 153.39 (d, *J*_CF_ = 238.0 Hz), 148.30, 145.36 (d, *J*_CF_ = 10.5 Hz), 136.22, 123.87 (d, *J*_CF_ = 6.6 Hz), 113.80 (d, *J*_CF_ = 22.9 Hz), 110.26, 103.85, 52.17, 51.36, 49.10, 46.09, 14.50. Anal. Calcd for C_17_H_20_FN_3_O_3_: C, 61.25; H, 6.05; N, 12.61. Found: C, 61.32; H, 6.06; N, 12.58.

### General Procedure for the Synthesis of Methyl Esters **3a** and **3b**

To a solution of the appropriate fluoroquinolone methyl ester (**2a** or **2b**) in anhydrous CH_3_CN (10 mL) at 40 °C was added 4-fluoro-1-nitro-2-(trifluoromethyl)benzene under stirring. The color of the solution immediately turned yellow. The temperature was raised up to 80 °C, and the reaction mixture was left under stirring overnight. The solvent was evaporated, and the crude was diluted with CH_2_Cl_2_ (50 mL) and washed with deionized water (3 × 25 mL). The organic phase was dried over Na_2_SO_4_, filtered, and reduced under vacuum. The residue was purified by flash chromatography using a CH_2_Cl_2_/CH_3_OH gradient eluting system. According to this procedure, the following products have been obtained.

#### Methyl 1-Cyclopropyl-6-fluoro-7-(4-(4-nitro-3-(trifluoromethyl)phenyl)piperazin-1-yl)-4-oxo-1,4-dihydroquinoline-3-carboxylate (**3a**)

The title compound was obtained using 0.265 g (0.77 mmol) of **2a** and 0.172 g (0.82 mmol) of 4-fluoro-1-nitro-2-(trifluoromethyl)benzene. Yellow solid (61%): mp 265–267 °C; ^1^H NMR (500 MHz, DMSO-*d*_6_): δ 8.44 (s, 1H), 8.12 (d, *J* = 9.5 Hz, 1H), 7.78 (d, *J* = 13.5 Hz, 1H), 7.47 (d, *J* = 7.5 Hz, 1H), 7.37 (d, *J* = 2.5 Hz, 1H), 7.31 (dd, *J* = 9.5, 2.5 Hz, 1H), 3.77–3.74 (m, 4H), 3.73 (s, 3H), 3.68–3.63 (m, 1H), 3.44–3.42 (m, 4H), 1.27–1.23 (m, 2H), 1.13–1.10 (m, 2H); ^13^C NMR (125 MHz, CDCl_3_): δ 173.12, 166.52, 153.44 (d, *J*_CF_ = 247.5 Hz), 153.15, 148.65, 143.88 (d, *J*_CF_ = 12.5 Hz), 138.15 (d, *J*_CF_ = 10.0 Hz), 128.70, 126.46 (d, *J*_CF_ = 33.8 Hz), 123.9 (d, *J*_CF_ = 7.5 Hz), 123.47, 121.29, 115.41, 113.91 (d, *J*_CF_ = 22.5 Hz), 112.72 (d, *J*_CF_ = 6.3 Hz), 110.56, 105.03, 52.30, 49.54, 47.29, 34.67, 8.36. Anal. Calcd. for C_25_H_22_F_4_N_4_O_5_: C, 56.18; H, 4.15; N, 10.48. Found: C, 56.31; H, 4.13; N, 10.51.

#### Methyl 1-Ethyl-6-fluoro-7-(4-(4-nitro-3-(trifluoromethyl)phenyl)piperazin-1-yl)-4-oxo-1,4-dihydroquinoline-3-carboxylate (**3b**)

The title compound was obtained using 0.500 g (1.5 mmol) of **2b** and 0.314 g (1.5 mmol) of 4-fluoro-1-nitro-2-(trifluoromethyl)benzene. Yellow solid (52%): mp 236–238 °C; ^1^H NMR (500 MHz, DMSO-*d*_6_): δ 8.65 (s, 1H), 8.12 (d, *J* = 9.0 Hz, 1H), 7.83 (d, *J* = 13.0 Hz, 1H), 7.37 (d, *J* = 2.5 Hz, 1H), 7.31 (dd, *J* = 9.5, 3.0 Hz, 1H), 7.08 (d, *J* = 7.0 Hz, 1H), 4.42 (q, *J* = 7.0 Hz, 2H), 3.76–3.74 (m, 7H), 3.43–3.41 (m, 4H), 1.38 (t, *J* = 7.5 Hz, 3H); ^13^C NMR (125 MHz, DMSO-*d*_6_): δ 171.46, 165.10, 153.28, 152.93, 151.31, 148.85, 143.63 (d, *J*_CF_ = 10.0 Hz), 136.16, 135.54, 128.83, 124.59, 123.54, 122.74 (d, *J*_CF_ = 6.3 Hz), 115.34, 111.92 (d, *J*_CF_ = 22.5 Hz), 111.72 (d, *J*_CF_ = 6.3 Hz), 109.15, 105.88, 51.16, 48.86, 48.05, 46.17, 14.23. Anal. Calcd. for C_24_H_22_F_4_N_4_O_5_: C, 55.17; H, 4.24; N, 10.72. Found: C, 55.28; H, 4.25; N, 10.75.

#### 2-((4-Nitro-3-(trifluoromethyl)phenyl)amino)ethanol (**4a**)

To a solution of 2-aminoethanol (1.00 g, 16.4 mmol) in anhydrous CH_3_CN (10 mL) was added 4-fluoro-1-nitro-2-(trifluoromethyl)benzene (2.32 g, 11.1 mmol). The reaction was left under stirring at 60 °C overnight. Then, the reaction solvent was removed under reduced pressure and the residue was dissolved in EtOAc and washed with a saturated solution of NaHCO_3_ (3 × 25 mL). The organic phase was dried over Na_2_SO_4_, filtered, and concentrated under vacuum. The crude was purified by flash chromatography eluting with a 20% of Cy in EtOAc to give the desired product. Yellow solid (72%): ^1^H NMR (500 MHz, CD_3_OD): δ 8.01 (d, *J* = 9.0 Hz, 1H), 7.04 (d, *J* = 2.5 Hz, 1H), 6.81 (dd, *J* = 9.0, 2.5 Hz, 1H), 3.74 (t, *J* = 5.5 Hz, 2H), 3.35 (t, *J* = 5.5 Hz, 2H); ^13^C NMR (125 MHz, CD_3_OD): δ 154.65, 136.41, 130.34, 127.27 (t, *J*_CF_ = 18.4 Hz), 125.08, 122.92, 113.05, 112.26, 61.15, 46.26. Anal. Calcd. for C_9_H_9_F_3_N_2_O_3_: C, 43.21; H, 3.63; N, 11.20. Found: C, 43.12; H, 3.64; N, 11.22.

#### 3-((4-Nitro-3-(trifluoromethyl)phenyl)amino)propan-1-ol (**4b**)

To a solution of 3-aminopropan-1-ol (0.143 g, 1.90 mmol) in anhydrous CH_3_CN (5 mL) was added 4-fluoro-1-nitro-2-(trifluoromethyl)benzene (0.200 g, 0.95 mmol). The reaction was left under stirring at 60 °C for 12 h in a closed glass Pyrex vial. After the reaction was complete, the reaction solvent was removed under reduced pressure and the residue was repeatedly triturated with *n*-hexane till the formation of a yellow solid. The solid was decanted, solubilized in EtOAc, and washed with a saturated solution of NaHCO_3_ (3 × 25 mL). The organic phase was dried over Na_2_SO_4_, filtered, and reduced under pressure, affording the pure desired product that did not require any further purification. Yellow solid (quantitative): ^1^H NMR (500 MHz, acetone-*d*_6_): δ 8.03 (d, *J* = 8.5 Hz, 1H), 7.09 (d, *J* = 3.0 Hz, 1H), 6.88 (dd, *J* = 6.5, 3.0 Hz, 1H), 6.67 (br s, 1H), 3.82–3.68 (m, 3H), 3.40 (q, *J* = 6.5 Hz, 2H), 1.90–1.85 (m, 2H); ^13^C NMR (125 MHz, acetone-*d*_6_): δ 154.19, 136.02, 130.26, 126.74 (t, *J*_CF_ = 38.3 Hz), 123.68 (d, *J*_CF_ = 270.8 Hz), 112.00 (d, *J*_CF_ = 145.5 Hz), 60.01, 40.97, 29.45. Anal. Calcd. for C_10_H_11_F_3_N_2_O_3_: C, 45.46; H, 4.20; N, 10.60. Found: 45.33; H, 4.21; N, 10.63.

### General Procedure for the Synthesis of Methanesulfonates **5a** and **5b**

To a solution of the appropriate alcohol (**4a** or **4b**) in anhydrous CH_2_Cl_2_ was added TEA at 0 °C. The reaction mixture was left under stirring for 30 min, and then methanesulfonyl chloride was added dropwise using a dropping funnel. The reaction was left under stirring at room temperature for 60 min. After this time, the solvent was removed under reduced pressure, the resulting residue was dissolved in CH_2_Cl_2_ and washed in sequence with a saturated solution of NH_4_Cl (25 mL) and a saturated solution of NaHCO_3_. The organic phase was dried over Na_2_SO_4_, filtered, and evaporated. The crude products were directly used in the next step with no further purification or characterization. According to this procedure, the following products have been synthesized.

#### 2-((4-Nitro-3-(trifluoromethyl)phenyl)amino)ethyl Methanesulfonate (**5a**)

The title compound was obtained using 0.100 g (0.4 mmol) of **4a** in 5 mL of anhydrous CH_2_Cl_2_, 166 μL (1.2 mmol) of TEA, and 62 μL (0.8 mmol) of methanesulfonyl chloride. Yellow oil (quantitative).

#### 3-((4-Nitro-3-(trifluoromethyl)phenyl)amino)propyl Methanesulfonate (**5b**)

The title compound was obtained using 0.300 g (1.13 mmol) of **4b** in 10 mL of anhydrous CH_2_Cl_2_, 0.345 g (3.40 mmol) of TEA, and 0.328 g (2.26 mmol) of methanesulfonyl chloride. Yellow oil (quantitative).

### General Procedure for the Synthesis of Methyl Esters **6a**–**6d**

In a two-neck round-bottom flask, the appropriate fluoroquinolone methyl ester (**2a** or **2b**) was added to the appropriate methanesulfonate solution (**5a** or **5b**) in anhydrous CH_3_CN under a N_2_ atmosphere. The reaction mixture was refluxed under stirring overnight. Then, the solvent was removed under reduced pressure, and a saturated solution of NaHCO_3_ was added to the residue. The aqueous phase was extracted with CH_2_Cl_2_ (3 × 25 mL), and then the organic phases were dried over Na_2_SO_4_, filtered, and concentrated under reduced pressure. The crude was purified by flash chromatography using a CH_2_Cl_2_/CH_3_OH gradient eluting system. According to this procedure, the following products have been obtained.

#### Methyl 1-Cyclopropyl-6-fluoro-7-(4-(2-((4-nitro-3-(trifluoromethyl)phenyl)amino)ethyl)piperazin-1-yl)-4-oxo-1,4-dihydroquinoline-3-carboxylate (**6a**)

The title compound was synthesized using 0.276 g (0.8 mmol) of **2a** in 15 mL of anhydrous CH_3_CN and 0.129 g (0.39 mmol) of **5a**. Yellow solid (69%): mp 231–233 °C; ^1^H NMR (500 MHz, DMSO-*d*_6_): δ 8.44 (s, 1H), 8.08 (d, *J* = 9.5 Hz, 1H), 7.75 (d, *J* = 13.0 Hz, 1H), 7.47 (t, *J* = 5.0 Hz, 1H), 7.43 (d, *J* = 7.5 Hz, 1H), 7.16 (s, 1H), 6.88 (dd, *J* = 9.0, 2.5 Hz, 1H), 3.73 (s, 3H), 3.66–3.62 (m, 1H), 3.37 (app. q, *J* = 6.0 Hz, 2H), 3.28–3.24 (m, 4H), 2.70–2.65 (m, 4H), 2.63 (t, *J* = 6.0 Hz, 2H), 1.26–1.24 (m, 2H), 1.11–1.08 (m, 2H); ^13^C NMR (125 MHz, DMSO-*d*_6_): δ 171.52, 164.95, 153.57, 152.38 (d, *J*_CF_ = 193.0 Hz), 148.22, 143.85 (d, *J*_CF_ = 9.6 Hz), 138.05, 133.55, 129.70, 124.82 (d, *J*_CF_ = 32.9 Hz), 123.63, 121.79 (d, *J*_CF_ = 6.1 Hz), 121.46, 111.50 (d, *J*_CF_ = 22.6 Hz), 108.98, 106.18, 55.97, 52.42, 51.23, 49.52, 34.71, 7.51. Anal. Calcd. for C_27_H_27_F_4_N_5_O_5_: C, 56.15; H, 4.71; N, 12.13. Found: C, 55.94; H, 4.70; N, 12.17.

#### Methyl 1-Cyclopropyl-6-fluoro-7-(4-(3-((4-nitro-3-(trifluoromethyl)phenyl)amino)propyl)piperazin-1-yl)-4-oxo-1,4-dihydroquinoline-3-carboxylate (**6b**)

The title compound was synthesized using 0.350 g (1.01 mmol) of **2a** in 15 mL of anhydrous CH_3_CN and 0.195 g (0.57 mmol) of **5b**. Yellow solid (50%): mp 202–204 °C; ^1^H NMR (500 MHz, DMSO-*d*_6_): δ 8.44 (s, 1H), 8.08 (d, *J* = 9.5 Hz, 1H), 7.75 (d, *J* = 13.5 Hz, 1H), 7.59 (t, *J* = 5.5 Hz, 1H), 7.43 (d, *J* = 7.5 Hz, 1H), 7.07 (s, 1H), 6.85 (dd, *J* = 9.5, 2.5 Hz, 1H), 3.73 (s, 3H), 3.67–3.62 (m, 1H), 3.29–3.23 (m, 6H), 2.61–2.56 (m, 4H), 2.46 (t, *J* = 7.0 Hz, 2H), 1.77 (m, 2H), 1.27–1.23 (m, 2H), 1.11–1.08 (m, 2H); ^13^C NMR (125 MHz, DMSO-*d*_6_): δ 171.53, 164.96, 152.42 (d, *J*_CF_ = 203.8 Hz), 148.22, 143.88 (d, *J*_CF_ = 10.0 Hz), 138.06, 133.38, 129.77, 125.02, 123.62, 121.77 (d, *J*_CF_ = 6.3 Hz), 121.45, 111.50 (d, *J*_CF_ = 22.5 Hz), 108.99, 106.16, 54.87, 52.48, 51.24, 49.57, 40.42, 34.70, 25.40, 7.51. Anal. Calcd. for C_28_H_29_F_4_N_5_O_5_: C, 56.85; H, 4.94; N, 11.84. Found: C, 57.01; H, 4.95; N, 11.81.

#### Methyl 1-Ethyl-6-fluoro-7-(4-(2-((4-nitro-3-(trifluoromethyl)phenyl)amino)ethyl)piperazin-1-yl)-4-oxo-1,4-dihydroquinoline-3-carboxylate (**6c**)

The title compound was synthesized using 0.400 g (1.2 mmol) of **2b** in 15 mL of anhydrous CH_3_CN and 0.197 g (0.6 mmol) of **5a**. Yellow solid (61%): mp 245–247 °C; ^1^H NMR (500 MHz, DMSO-*d*_6_): δ 8.64 (s, 1H), 8.08 (d, *J* = 9.5 Hz, 1H), 7.79 (d, *J* = 13.5 Hz, 1H), 7.47 (t, *J* = 5.0 Hz, 1H), 7.15 (s, 1H), 7.03 (d, *J* = 7.0 Hz, 1H), 6.88 (dd, *J* = 9.5, 2.5 Hz, 1H), 4.40 (q, *J* = 7.0 Hz, 2H), 3.73 (s, 3H), 3.37 (q, *J* = 6.0 Hz, 2H), 3.27–3.25 (m, 4H), 2.67–2.65 (m, 4H), 2.62 (t, *J* = 6.0 Hz, 2H), 1.37 (t, *J* = 7.0 Hz, 3H); ^13^C NMR (125 MHz, DMSO-*d*_6_): δ 171.46, 165.11, 153.40, 152.29 (d, *J*_CF_ = 213.5 Hz), 148.78, 144.16 (d, *J*_CF_ = 10.3 Hz), 136.16, 133.53, 129.71, 124.81 (d, *J*_CF_ = 31.9 Hz), 123.62, 122.54 (d, *J*_CF_ = 6.3 Hz), 121.45, 111.81 (d, *J*_CF_ = 22.4 Hz), 109.10, 105.69, 55.96, 52.45, 51.14, 49.54, 48.02, 14.19. Anal. Calcd. for C_26_H_27_F_4_N_5_O_5_: C, 55.22; H, 4.81; N, 12.38. Found: C, 55.40; H, 4.80; N, 12.40.

#### Methyl 1-Ethyl-6-fluoro-7-(4-(3-((4-nitro-3-(trifluoromethyl)phenyl)amino)propyl)piperazin-1-yl)-4-oxo-1,4-dihydroquinoline-3-carboxylate (**6d**)

The title compound was synthesized using 0.465 g (1.4 mmol) of **2b** in 15 mL of anhydrous CH_3_CN and 0.240 g (0.7 mmol) of **5b**. Yellow solid (50%): mp 216–218 °C; ^1^H NMR (500 MHz, DMSO-*d*_6_): δ 8.64 (s, 1H), 8.08 (d, *J* = 9.5 Hz, 1H), 7.78 (d, *J* = 13.5 Hz, 1H), 7.59 (t, *J* = 5.5 Hz, 1H), 7.07 (s, 1H), 7.03 (d, *J* = 7.0 Hz), 6.84 (dd, *J* = 9.0, 2.0 Hz, 1H), 4.40 (q, *J* = 7.0 Hz, 2H), 3.73 (s, 3H), 3.28–3.20 (m, 6H), 2.60–2.54 (br m, 4H), 2.45 (t, *J* = 7.0 Hz, 2H), 1.77 (m, 2H), 1.37 (t, *J* = 7.0 Hz, 3H); ^13^C NMR (125 MHz, DMSO-*d*_6_): δ 171.47, 165.11, 153.40, 152.33 (d, *J*_CF_ = 223.4 Hz), 148.78, 144.18 (d, *J*_CF_ = 10.3 Hz), 136.16, 133.37, 129.81, 125.00, 123.61, 122.52 (d, *J*_CF_ = 6.3 Hz), 121.44, 111.79 (d, *J*_CF_ = 22.5 Hz), 109.10, 105.66, 54.88, 52.50, 51.14, 49.60, 48.01, 40.43, 25.39, 14.19. Anal. Calcd. for C_27_H_29_F_4_N_5_O_5_: C, 55.96; H, 5.04; N, 12.08. Found: C, 56.08; H, 5.05; N, 12.06.

### General Procedure for the Synthesis of Carboxylic Acids **7a**–**7d**

In a round-bottom flask, the appropriate methyl ester (**6a**–**6d**) and a solution of 2 M NaOH were refluxed for 24 h under vigorous stirring. After this time, the reaction mixture was cooled, and a solution of 2 M HCl was added up to the isoelectric point. The obtained precipitate was filtered under vacuum and washed in sequence with deionized water, isopropanol, and diethyl ether. The solid was then purified by flash chromatography using a CH_2_Cl_2_/CH_3_OH gradient eluting system. According to this procedure, the following products have been obtained.

#### 1-Cyclopropyl-6-fluoro-7-(4-(2-((4-nitro-3-(trifluoromethyl)phenyl)amino)ethyl)piperazin-1-yl)-4-oxo-1,4-dihydroquinoline-3-carboxylic Acid (**7a**)

The title compound was obtained starting from 0.159 g (0.28 mmol) of **6a** and 25 mL of 2 M NaOH. Yellow solid (75%): mp 229–231 °C; ^1^H NMR (500 MHz, DMSO-*d*_6_): δ 8.59 (s, 1H), 8.08 (d, *J* = 9.0 Hz, 1H), 7.83 (d, *J* = 13.5 Hz, 1H), 7.52–7.47 (m, 2H), 7.16 (s, 1H), 6.89 (d, *J* = 8.5 Hz, 1H), 3.70 (br s, 1H), 3.29 (m, 6H), 2.70–2.65 (br s, 4H), 2.63 (t, *J* = 5.5 Hz, 2H), 1.28–1.27 (br m, 2H), 1.09 (br s, 2H); ^13^C NMR (125 MHz, DMSO-*d*_6_): δ 175.45, 166.42, 153.18, 147.48, 146.44, 138.66, 133.53, 129.73, 124.96, 124.71, 123.65, 119.32, 111.11 (d, *J*_CF_ = 21.3 Hz), 105.87, 55.97, 53.84, 52.43, 49.53, 40.11, 7.53. Anal. Calcd. for C_26_H_25_F_4_N_5_O_5_: C, 55.42; H, 4.47; N, 12.43. Found: C, 55.61; H, 4.48; N, 12.40.

#### 1-Cyclopropyl-6-fluoro-7-(4-(3-((4-nitro-3-(trifluoromethyl)phenyl)amino)propyl)piperazin-1-yl)-4-oxo-1,4-dihydroquinoline-3-carboxylic Acid (**7b**)

The title compound was obtained starting from 0.112 g (0.19 mmol) of **6b** and 25 mL of 2 M NaOH. Yellow solid (74%): mp 235–237 °C; ^1^H NMR (500 MHz, DMSO-*d*_6_): δ 8.66 (s, 1H), 8.08 (d, *J* = 9.0 Hz, 1H), 7.90 (d, *J* = 13.0 Hz, 1H), 7.63 (s, 1H), 7.57 (d, *J* = 7.0 Hz, 1H), 7.08 (s, 1H), 6.86 (dd, *J* = 9.0, 2.0 Hz, 1H), 3.86–3.80 (br m, 1H), 3.41–3.33 (br s, 4H), 3.29–3.25 (m, 2H), 2.61 (br s, 4H), 2.48–2.42 (br m overlapped with DMSO, 2H), 1.79 (br s, 2H), 1.33–1.30 (m, 2H), 1.20–1.17 (m, 2H); ^13^C NMR (125 MHz, DMSO-*d*_6_): δ 176.32, 165.88, 153.96, 152.59 (d, *J*_CF_ = 152.5 Hz), 147.99, 139.15, 133.40, 129.79, 124.8 (d, *J*_CF_ = 32.5 Hz), 123.61, 121.44, 118.57, 110.94 (d, *J*_CF_ = 23.8 Hz), 106.73, 106.33, 54.88, 52.25, 50.63, 40.34, 35.84, 28.99, 7.55. Anal. Calcd. for C_27_H_27_F_4_N_5_O_5_: C, 56.15; H, 4.71; N, 12.13. Found: C, 56.02; H, 4.70; N, 12.15.

#### 1-Ethyl-6-fluoro-7-(4-(2-((4-nitro-3-(trifluoromethyl)phenyl)amino)ethyl)piperazin-1-yl)-4-oxo-1,4-dihydroquinoline-3-carboxylic Acid (**7c**)

The title compound was obtained starting from 0.177 g (0.31 mmol) of **6c** and 25 mL of 2 M NaOH. Yellow solid (62%): mp 225–227 °C; ^1^H NMR (500 MHz, DMSO-*d*_6_): δ 8.94 (s, 1H), 8.07 (d, *J* = 9.0 Hz, 1H), 7.91 (d, *J* = 13.5 Hz, 1H), 7.47 (t, *J* = 5.5 Hz, 1H), 7.21–7.14 (m, 2H), 6.88 (dd, *J* = 9.5, 2.0 Hz, 1H), 4.58 (q, *J* = 7.0 Hz, 2H), 3.39–3.33 (m, 6H), 2.69–2.65 (m, 4H), 2.63 (t, *J* = 6.0 Hz, 2H), 1.41 (t, *J* = 7.0 Hz, 3H); ^13^C NMR (125 MHz, DMSO-*d*_6_): δ 176.12, 166.08, 153.84, 152.50 (d, *J*_CF_ = 160.0 Hz), 148.47, 145.42 (d, *J*_CF_ = 10.0 Hz), 137.18, 133.54, 129.71, 124.82 (d, *J*_CF_ = 32.5 Hz), 123.63, 121.46, 119.19 (d, *J*_CF_ = 7.5 Hz), 111.15 (d, *J*_CF_ = 22.5 Hz), 107.06, 105.76, 55.93, 52.37, 49.45, 49.42, 49.03, 14.29. Anal. Calcd. for C_25_H_25_F_4_N_5_O_5_: C, 54.45; H, 4.57; N, 12.70. Found: C, 54.31; H, 4.56; N, 12.66.

#### 1-Ethyl-6-fluoro-7-(4-(3-((4-nitro-3-(trifluoromethyl)phenyl)amino)propyl)piperazin-1-yl)-4-oxo-1,4-dihydroquinoline-3-carboxylic Acid (**7d**)

The title compound was obtained starting from 0.077 g (0.13 mmol) of **6d** and 25 mL of 2 M NaOH. Yellow solid (36%): mp 249–251 °C; ^1^H NMR (500 MHz, DMSO-*d*_6_): δ 8.94 (s, 1H), 8.07 (d, *J* = 9.0 Hz, 1H), 7.91 (d, *J* = 13.5 Hz, 1H), 7.59 (s, 1H), 7.17 (s, 1H), 7.06 (s, 1H), 6.84 (br m,1H), 4.59 (app s, 2H), 3.29–3.21 (br m, 6H), 2.62–2.53 (br m, 4H), 2.46 (t, *J* = 6.0 Hz, 2H), 1.81–1.73 (br m, 2H), 1.42 (t, *J* = 7.0 Hz, 3H); ^13^C NMR (125 MHz, DMSO-*d*_6_): δ 176.14, 166.04, 153.77, 152.44 (d, *J*_CF_ = 163.8 Hz), 148.59, 137.16, 133.55, 129.76, 126.58, 125.47, 124.85 (d, *J*_CF_ = 31.3 Hz), 123.60, 121.43, 119.26, 111.27 (d, *J*_CF_ = 22.5 Hz), 107.12, 69.77, 49.07, 33.65, 31.26, 28.98, 22.06, 14.38. Anal. Calcd. for C_26_H_27_F_4_N_5_O_5_: C, 55.22; H, 4.81; N, 12.38. Found: C, 55.07; H, 4.82; N, 12.35.

### Griess Test

The stock solution of the Griess reagent (purchased by Sigma Aldrich Srl) was obtained by dissolving 200 mg of the powder in 5 mL of H_2_O MilliQ. For nitrite quantification, each selected compound was dissolved in anhydrous DMSO to obtain a 1 mM solution (A). Subsequently, 160 μL of each solution A was diluted with 840 μL of H_2_O MilliQ to obtain solution B (160 μM). A total of 500 μL of B was then added to a quartz cuvette containing 500 μL of Griess reagent solution (80 μM). The resulting solutions were then irradiated with a 300 W tungsten lamp at a distance of 40 cm for 15 min, 1 h, and 2 h, recording the absorbance spectrum of each sample at each time point and reading the peak increase at 540 nm. The relationship of absorbance and concentrations of nitrite was constructed by drawing a standard curve using the known concentrations of NaNO_2_ (5, 10, 20, 30, 40, and 50 μM).

### Cell Lines and *In Vitro* Culture Conditions

The cell lines DU145 (HTB-81, human prostate carcinoma), PC3 (CRL-1435, human prostate adenocarcinoma), MCF7 (HTB-22, human breast adenocarcinoma), MDA-MB231 (HTB-26, human breast adenocarcinoma), and HCT116 (CCL-247, human colorectal carcinoma) were obtained from ATCC (American Type Culture Collection, Manassas, VA, USA); WH1 human fibroblasts were kindly provided by Dr. Guven.^51^ All the cells were maintained under standard culture conditions (37 °C; 5% CO_2_) in the RMPI1640 medium (PC3, MCF7, and MDA-MB231 cells), while DU145 and HCT116 cells were in the DMEM and Iscove’s medium (WH1 cells) supplemented with 10% fetal calf serum, 1% glutamine, and 1% antibiotics mixture; for HCT116 and DU145 cells, 1% sodium pyruvate and 1% non-essential amino acids were also added to the culture medium.

### Cell Viability Assay

The 3-(4,5-dimethylthiazol-2-yl)-2,5-diphenyltetrazolium bromide (MTT) assay was performed on all the cell lines tested as previously described^52^ with minor modifications. Briefly, according to the growth profiles previously defined for each cell line, adequate numbers of cells were plated in each well of a 96-well plate in 0.1 mL of complete culture medium. Cells were allowed to attach for 24 h before the treatment at 37 °C for 72 h with the compounds at concentrations ranging between 0.1 and 75 μM, bringing the final volume to 0.2 mL/well. Each experiment included eight replications per concentration tested; control samples were run with 0.2% DMSO. At the end of the incubation period, MTT (0.05 mL of a 2 mg/mL stock solution in PBS) was added to each well for 3 h at 37 °C. Cell supernatants were then carefully removed, the blue formazan crystals formed through MTT reduction by metabolically active cells were dissolved in 0.120 mL of DMSO, and the corresponding optical densities were measured at 570 nm using a Universal Microplate Reader EL800 (Bio-Tek, Winooski, VT).

To evaluate the contribution of NO release on cell viability, the MTT assay was performed on DU145 and PC3 cells following treatment with **7b** and **7c** with 1 h irradiation and 48 h incubation in a drug-free medium.

IC_50_ values were estimated from the resulting concentration–response curves by nonlinear regression analysis using GraphPad Prism software, v. 5.0 (GraphPad, San Diego, CA, USA). Differences between IC_50_ values were evaluated statistically by analysis of variance with a Bonferroni post-test for multiple comparisons.

### Minimum Inhibitory Concentration Determination

*P. aeruginosa* PAO1 was chosen as a model microorganism^53^ and was grown overnight in a Luria Bertani (LB) medium at 37 °C on an orbital shaker at 200 rpm. Minimum inhibitory concentrations (MICs) of quinolone and quinolone derivatives were determined against *P. aeruginosa* PAO1 by a broth dilution method. Overnight cultures were diluted 100-fold to give a cellular concentration of 10^7^ CFU/mL. Decreasing concentrations of compounds, from 200 to 0.1 μg/mL, were added to bacterial samples in a two-fold dilution series. Upon 24 h of incubation at 37 °C, the bacterial samples were observed for microbial growth, and MIC values were determined as minimal concentrations of drugs at which no turbidity was detectable. The assays were performed at least three times.

### Bacterial Binding Assay

The relative efficiency of fluoroquinolone and derivatives in binding *P. aeruginosa* cells was determined by an indirect method.^54^*P. aeruginosa* PAO1 overnight cultures were centrifuged (5000 rpm for 10 min), and the supernatant was removed. Pellets were suspended and 10-fold diluted in PBS. Fluoroquinolone and fluoroquinolone derivatives were administered at 10 μM and incubated for 1 h at 37 °C in the dark to allow the interaction between the compounds and cells. After dark incubation, samples were centrifuged (10,000 rpm for 10 min), and the visible spectra of the supernatant were recorded (*k* = 380–700 nm) and compared with the corresponding visible spectrum of each compound.

### Antimicrobial Photo-Inactivation Test

Upon the overnight growth of *P. aeruginosa* PAO1 and *S. aureus* ATCC6538P (methicillin susceptible *S. aureus*, MSSA), cultures were diluted in phosphate buffer (PBS–KH_2_PO_4_/K_2_HPO_4_, 10 mM, pH 7.4) to reach a concentration of ∼10^6^ CFU/mL. Compound **7c** was added to a cell suspension at a final concentration of 10 μM. Cells were incubated in the dark for 10 min and then irradiated under light at 410 ± 10 nm (20 J/cm^2^) or incubated in the dark as a control. Soon after irradiation, the bacterial concentration was evaluated by the viability count technique. Briefly, an aliquot of each sample was 10-fold serially diluted in PBS and a volume of 10 μL of each diluted and undiluted sample was inoculated on LB agar. After overnight incubation at 37 °C, the corresponding cellular concentration was calculated and expressed as CFU/mL.

### Molecular Docking

Flexible ligand docking experiments were performed employing AutoDock 4.2.6 software implemented in YASARA (v. 19.5.5, YASARA Biosciences GmbH, Vienna, Austria)^55,56^ using the crystal structure of the human Topo IIα (PDB ID: 5GWK), bacterial Topo IIA (PDB ID: 2XCT), and bacterial DNA gyrase (PDB ID: 6M1S) retrieved from the PDB Data Bank as a fully optimized one and the Lamarckian genetic algorithm (LGA). The maps were generated by the program AutoGrid (4.2.6) with a spacing of 0.375 Å and dimensions that encompass all atoms extending 5 Å from the surface of the structure of the crystallized ligands. All the parameters were inserted at their default settings as previously reported.^57^ In the docking tab, the macromolecule and ligand are selected, and GA parameters are set as ga_runs = 100, ga_pop_size = 150, ga_num_evals = 25,000,000, ga_num_generations = 27,000, ga_elitism = 1, ga_mutation_rate = 0.02, ga_crossover_rate = 0.8, ga_crossover_mode = two points, ga_cauchy_alpha = 0.0, ga_cauchy_beta = 1.0, and number of generations for picking worst individual = 10. Since no water molecules are directly involved in complex stabilization, they were not considered in the docking process (although in the crystallized structure of the bacterial DNA gyrase there are three structural water molecules that form hydrogen bonds between the ligand and some of the amino acids present on the enzymatic site, this network of H-bonds is intrinsic with the structure of the crystallized ligand. Docking calculations performed with the crystallized water molecules led to unsatisfactory results; therefore, the removal of water molecules in the peripheral regions of the binding site does not influence the calculated free binding energies in any way). All protein amino acid residues were kept rigid, whereas all single bonds of ligands were treated as fully flexible. The values of the energies of docking, in kcal/mol, have been calculated employing the “hybrid” force field implemented in AutoDock that contains terms based on molecular mechanics as well as empirical. Although the prediction of absolute binding energies may be less accurate compared to more computationally expensive, purely force field-based methods, this semi-empirical approach is considered as well-suited for the relative rankings.^58^

### Molecular Optimization

The semi-empirical calculations were performed using the parameterized model number 6 Hamiltonian^59^ as implemented in the MOPAC package (MOPAC2016 v. 18.151, Stewart Computational Chemistry, Colorado Springs, Colorado, USA). All molecules were fully optimized employing the eigenvector following the algorithm and a gradient minimization of 0.01 together with the precise and ddmin = 0 keywords.

### Molecular Dynamics Simulations

The MD simulations of the human Topo IIα/DNA/**1a** ternary model system were performed with the YASARA Structure package (19.11.5).^55^ A periodic simulation cell with boundaries extending 8 Å from the surface of the complex was employed. The box was filled with water, with a maximum sum of all bump water of 1.0 Å and a density of 0.997 g/mL with an explicit solvent. YASARA’s p*K*_a_ utility was used to assign p*K*_a_ values at pH 7.4,^60^ and system charges were neutralized with NaCl (0.9% by mass). Water molecules were deleted to readjust the solvent density to 0.997 g/mL. The final system dimensions were approximately 122 × 122 × 122 Å^3^. The ligand force-field parameters were generated with the AutoSMILES utility,^57^ which employs semi-empirical AM1 geometry optimization. Moreover, the assignment of charges, by the assignment of the AM1BCC atom and bond types with refinement was performed using the RESP charges, and finally the assignments of general AMBER force field atom types. Optimization of the hydrogen bond network of the various enzyme–ligand complexes was obtained using the method established by Hooft *et al*.^61^ This model allowed addressing ambiguities arising from multiple side-chain conformations and protonation states that are not well resolved in the electron density. The protein was treated with an AMBER ff14SB force field and^62^ the ligand with GAFF2,^63^ and the TIP3P model was used for water. The cutoff was 8 Å for van der Waals forces (the default used by AMBER),^64^ and no cutoff was applied to electrostatic forces (using the Particle Mesh Ewald algorithm).^65^ A 100 ps MD simulation was run on the solvent only. The entire system was then energy-minimized using first the steepest descent minimization to remove conformational stress followed by a simulated annealing minimization until convergence (<0.01 kcal/mol Å). The equations of motions were integrated with multiple timesteps of 1.25 fs for bonded interactions and 2.5 fs for nonbonded interactions using the NPT ensemble at a temperature of 298 K and a pressure of 1 atm. The temperature was controlled using the Berendsen thermostat,^66^ and the pressure was controlled using the solvent-probe pressure control mode barostat.^67^ The MD simulation was then initiated with an equilibration period of 10 ns for the assessment of the ligand’s correct pose, and a classical production MD simulation of 100 ns was performed analogously to other experiments reported by us.^68,69^ The MD trajectories were recorded every 100 ps.

### MM/PBSA Calculation of the Energies of Binding during the MD Simulation

To this purpose, we used the iPBSA script, according to the procedure reported in detail in the original publication,^70^ with the algorithm for MM/PBSA implemented in the freely available AmberTools21 suite,^71^ to analyze the ligand/enzyme complex coordinates recorded during the MD simulation.

## Supporting Information Available

NMR spectra, fluorescence and absorption spectra, visible spectrum before and after cell binding, and IC_50_ values on DU145, PC3, MCF7, MDA-MB231, and HCT116 cell lines of compounds **1a**, **1b**, **3a**, **3b**, **6a**–**6d**, and **7a**–**7d** (PDF)
Molecular formula strings (CSV)

## ABBREVIATIONS

aPSs: antimicrobial photosensitizers
CFU: colony forming unit
Cip: Ciprofloxacin
DOX: doxorubicin
Gyr: gyrase
MD: molecular dynamics
MDR: multidrug resistance
MIC: minimal inhibitory concentration
NO: nitric oxide
Nor: Norfloxacin
PBS: phosphate buffered saline
*p*-TsOH: *para*-toluenesulfonic acid
R.I.: resistance index
TEA: triethylamine
TLC: thin-layer chromatography
TMS: tetramethylsilane
Topo: topoisomerase
