# Correction to “Nitric Oxide Photo-Donor Hybrids of Ciprofloxacin and Norfloxacin: A Shift in Activity from Antimicrobial to Anticancer Agents”

**DOI:** 10.1021/acs.jmedchem.2c01209

**Published:** 2022-08-10

**Authors:** Antonino Nicolò Fallica, Carla Barbaraci, Emanuele Amata, Lorella Pasquinucci, Rita Turnaturi, Maria Dichiara, Sebastiano Intagliata, Marzia Bruna Gariboldi, Emanuela Marras, Viviana Teresa Orlandi, Claudia Ferroni, Cecilia Martini, Antonio Rescifina, Davide Gentile, Greta Varchi, Agostino Marrazzo

Pages 11611 and 11612, references 18 and 42 should be switched, such that ref 18, cited on page 11598, refers to the work by Sorrenti et al., and ref 42, cited on page 11599, refers to the work by Amata et al.

Page 11599. A new reference (cited here as ref (1)) should be added at the end of the first sentence of the last paragraph of the Introduction: “...endowed with a 4-nitro-3-trifluoromethylaniline moiety for the light-triggered release of NO.”
