# Supplementary material for: Nitric Oxide Photo-Donor Hybrids of Ciprofloxacin and Norfloxacin: A Shift in Activity from Antimicrobial to Anticancer Agents
Source: J Med Chem. 2021 Jul 28;64(15):11597–613. doi: 10.1021/acs.jmedchem.1c00917 (PMC8389907; doi:10.1021/acs.jmedchem.1c00917)
Supplement: Supplementary file 1 — jm1c00917_si_001.pdf [file jm1c00917_si_001.pdf]

Supporting information

Nitric Oxide Photo-donor Hybrids of Ciprofloxacin and Norfloxacin: Shift in Activity from Antimicrobial to Anticancer Agents

Antonino Nicolò Fallica,[a] Carla Barbaraci,[a] Emanuele Amata,[a] Lorella Pasquinucci,[a] Rita Turnaturi,[a] Maria Dichiara,[a] Sebastiano Intagliata,[a] Marzia Bruna Gariboldi,[b] Emanuela Marras,[b] Viviana Teresa Orlandi,[b] Claudia Ferroni,[c] Cecilia Martini,[c] Antonio Rescifina,[a] Davide Gentile,[a] Greta Varchi,[c*] Agostino Marrazzo.[a*]

[a]Department of Drug and Health Sciences (DSFS) – University of Catania, Viale A. Doria, 6, 95125 Catania, Italy

[b]Department of Biotechnology and Life Sciences (DBSV) - University of Insubria, Via JH Dunant 3, 21100 Varese, Italy

[c]Institute for the Organic Synthesis and Photoreactivity – ISOF- Via Piero Gobetti, 101, 40129 Bologna, Italy

Corresponding author

*Email: marrazzo@unict.it;

*Email: greta.varchi@isof.cnr.it;

Table of contents

NMR spectra of compounds **1a**,**b**, **3a**,**b**, **6a**–**d** and **7a**–**d**.	S2–S13
**Figure S25.** Fluorescence and Absorption spectrum of compounds **1a**,**b**, **3a**,**b**, **6a**–**d** and **7a**–**d**.	S14
**Figure S26.** Visible spectrum of **1b**, **3b**, **6c**,**d** and **7c**,**d** before and after cell binding.	S15
**Figure S27.** Visible spectrum of **1a**, **3a**, **6a**,**b** and **7a**,**b** before and after cell binding.	S16
**Table S1.** IC_{50} Values of Compounds **1a**, **3a**, **6a**,**b** and **7a**,**b** on DU145, PC3, MCF7, MDA-MB231 and HCT116 Cell Lines	S17
**Table S2.** IC_{50} Values of Compounds **1b**, **3b**, **6c**,**d** and **7c**,**d** on DU145, PC3, MCF7, MDA-MB231 and HCT116 Cell Lines	S17
**Figure S28.** Variation of the energy of binding along the MD simulation trajectory for compound **1a**.	S18

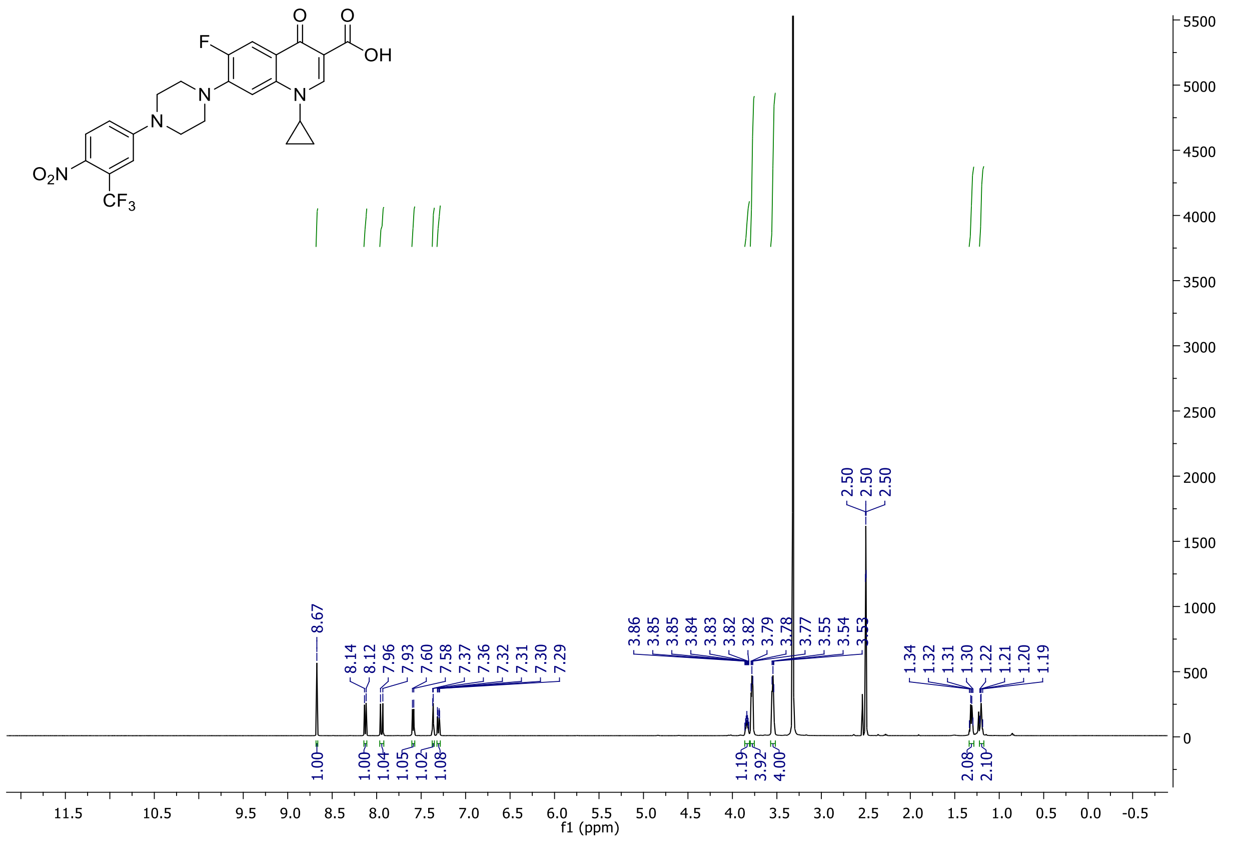

Figure S1. ^{1}H NMR (500 MHz, DMSO-*d6*) of compound **1a**.

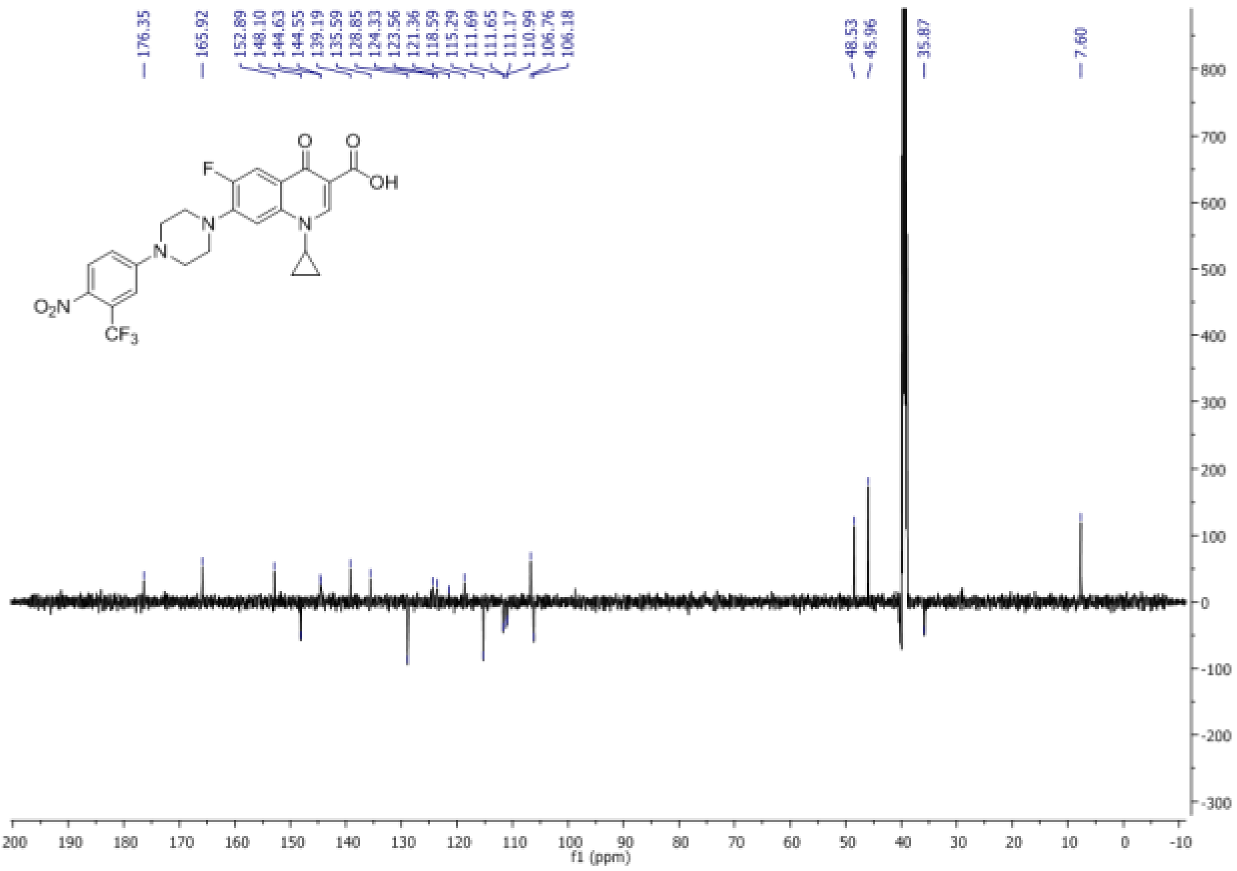

Figure S2. ^{13}C NMR (125 MHz, DMSO-*d6*) of compound **1a**.

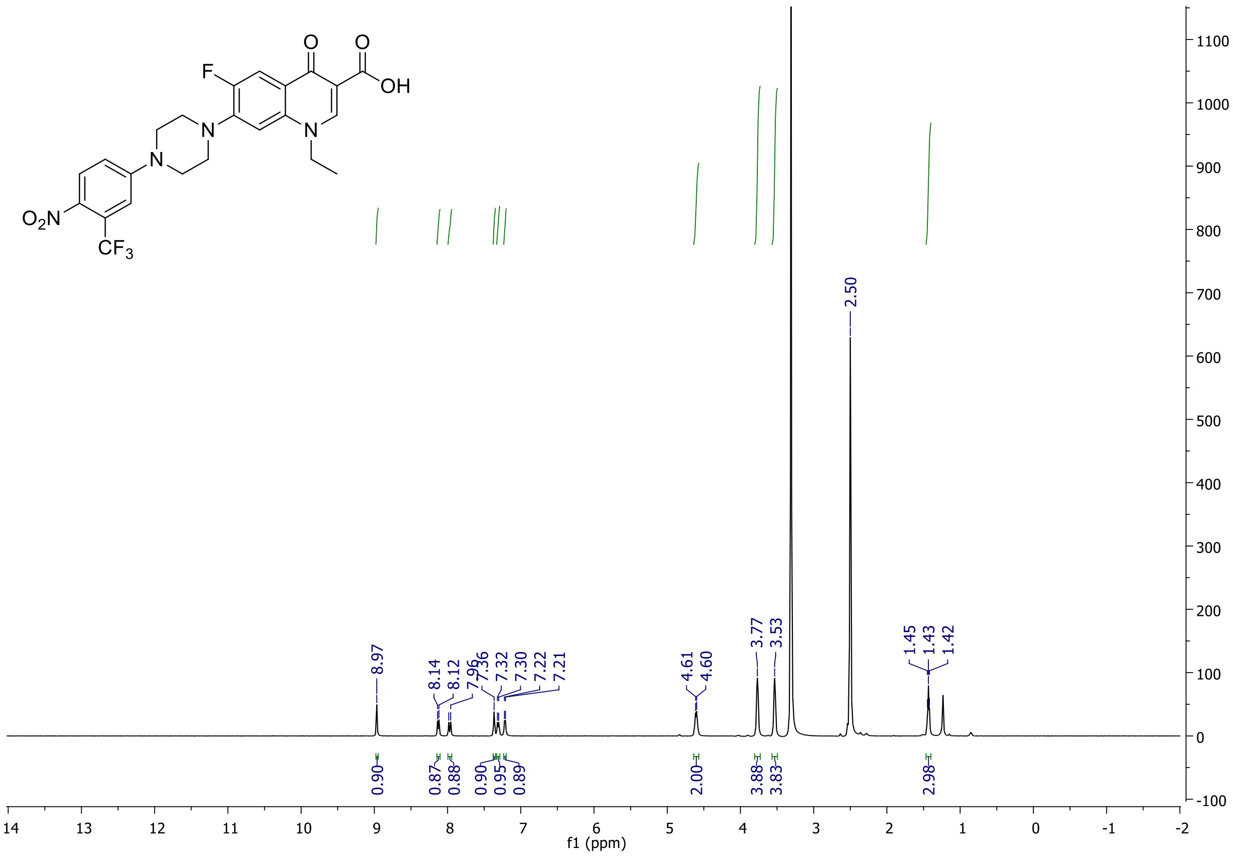

Figure S3. ^{1}H NMR (500 MHz, DMSO-*d6*) of compound **1b**.

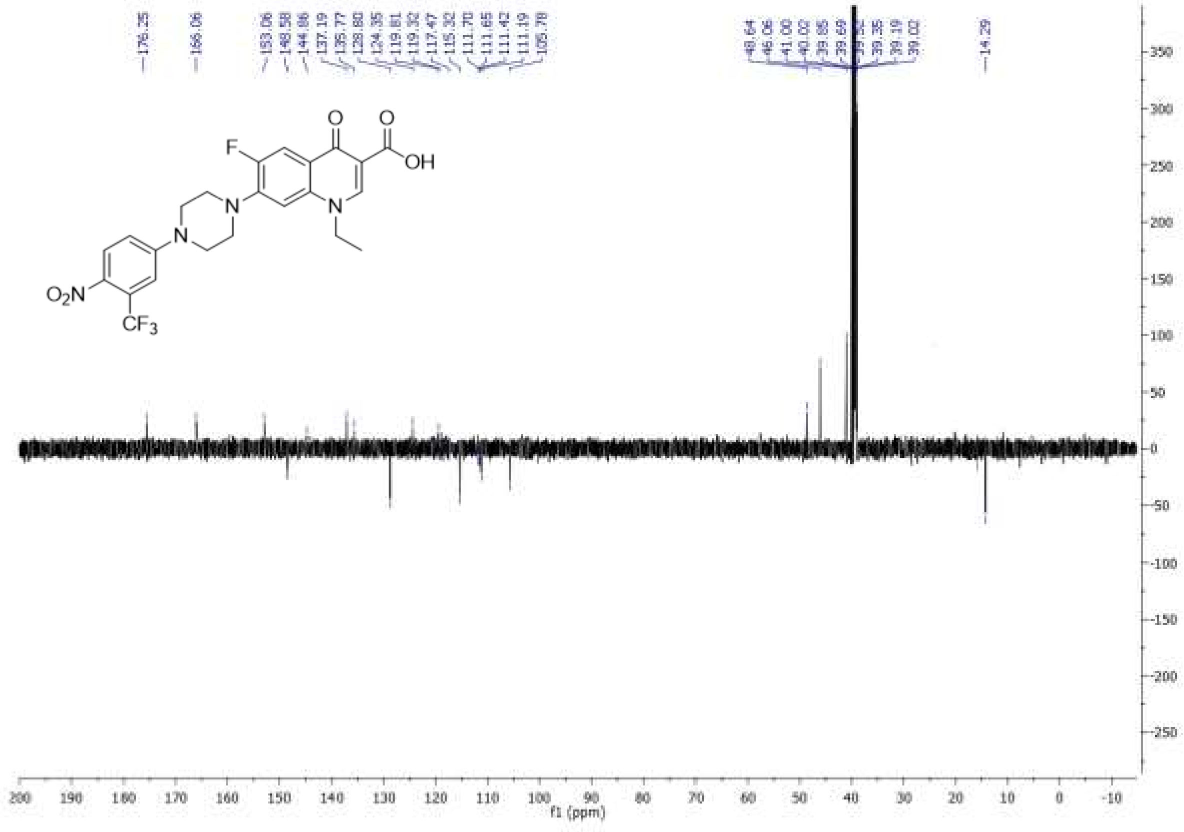

Figure S4. ^{13}C NMR (125 MHz, DMSO-*d6*) of compound **1b**.

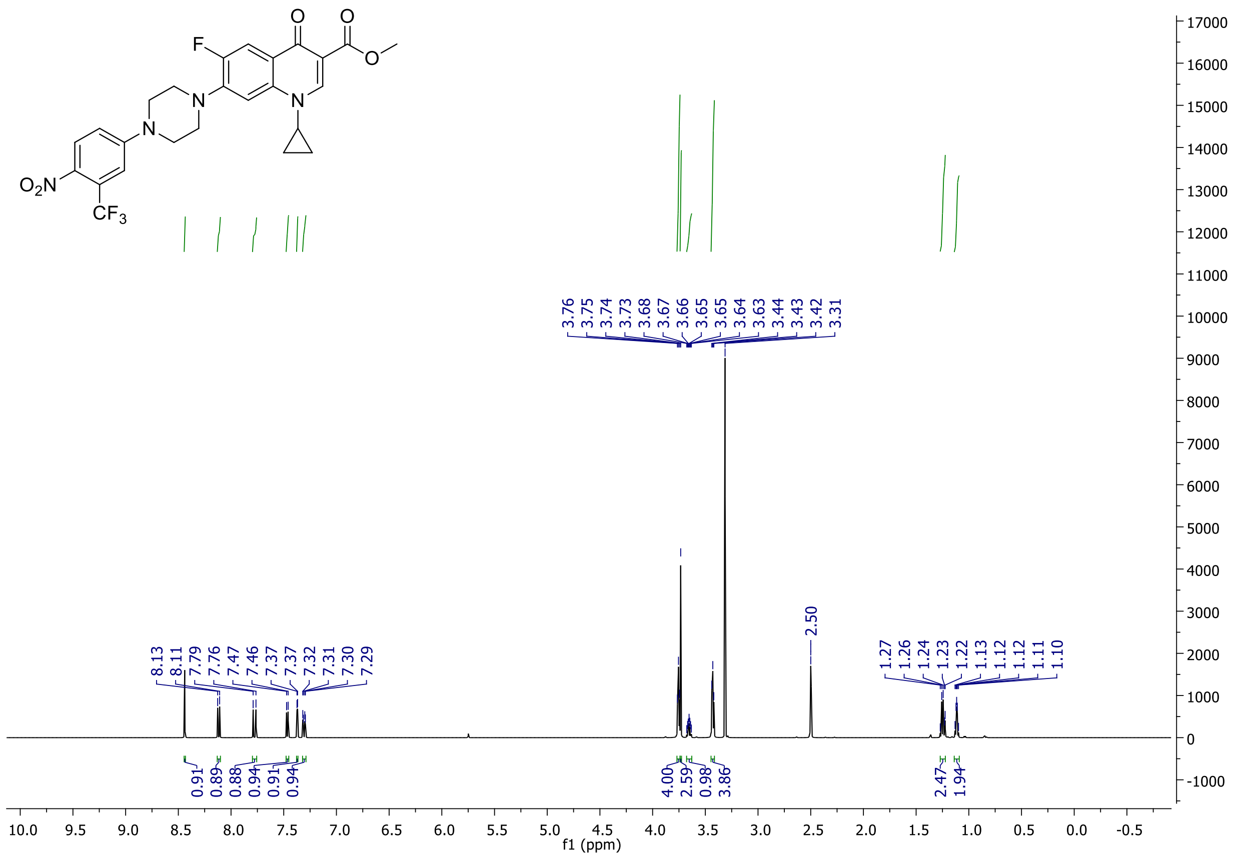

Figure S5. ^{1}H NMR (500 MHz, DMSO-*d6*) of compound **3a**.

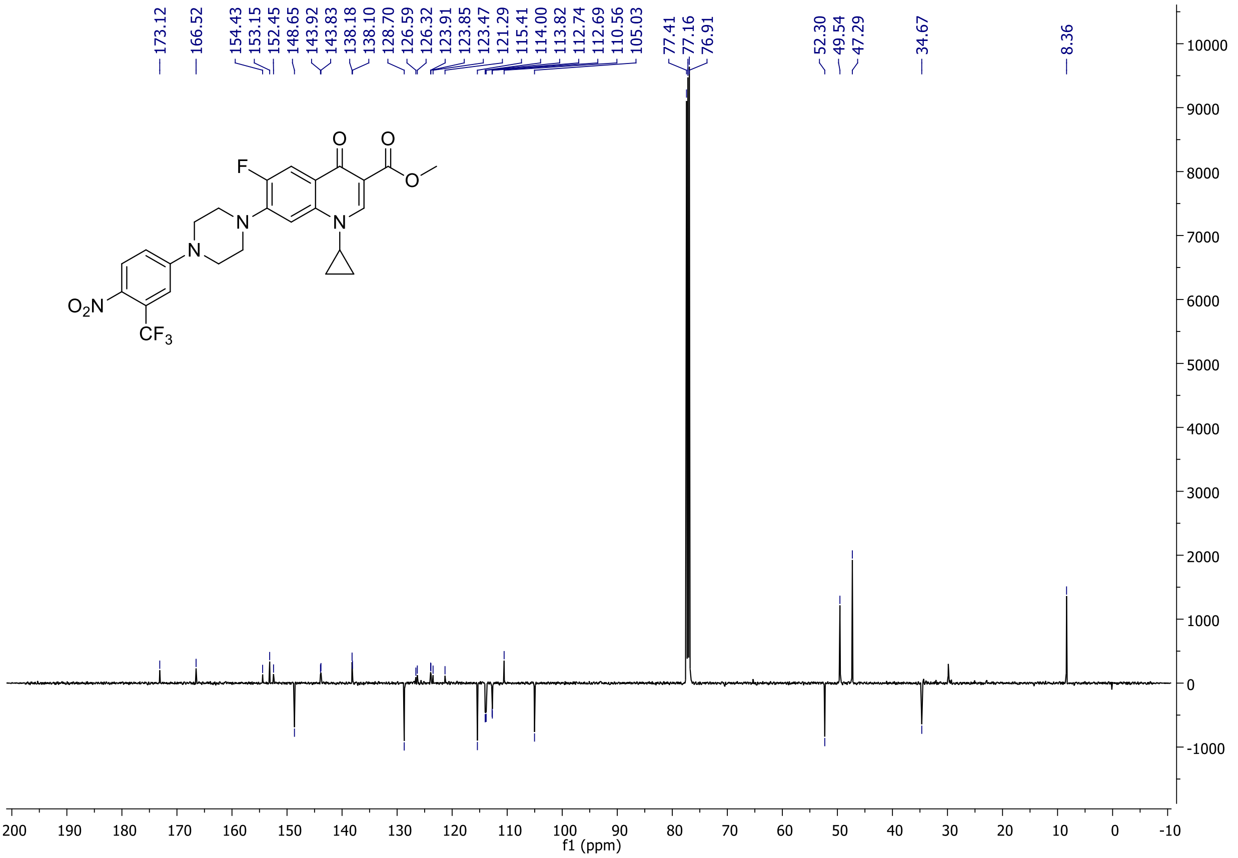

Figure S6. ^{13}C NMR (125 MHz, $CDCl_3$) of compound **3a**.

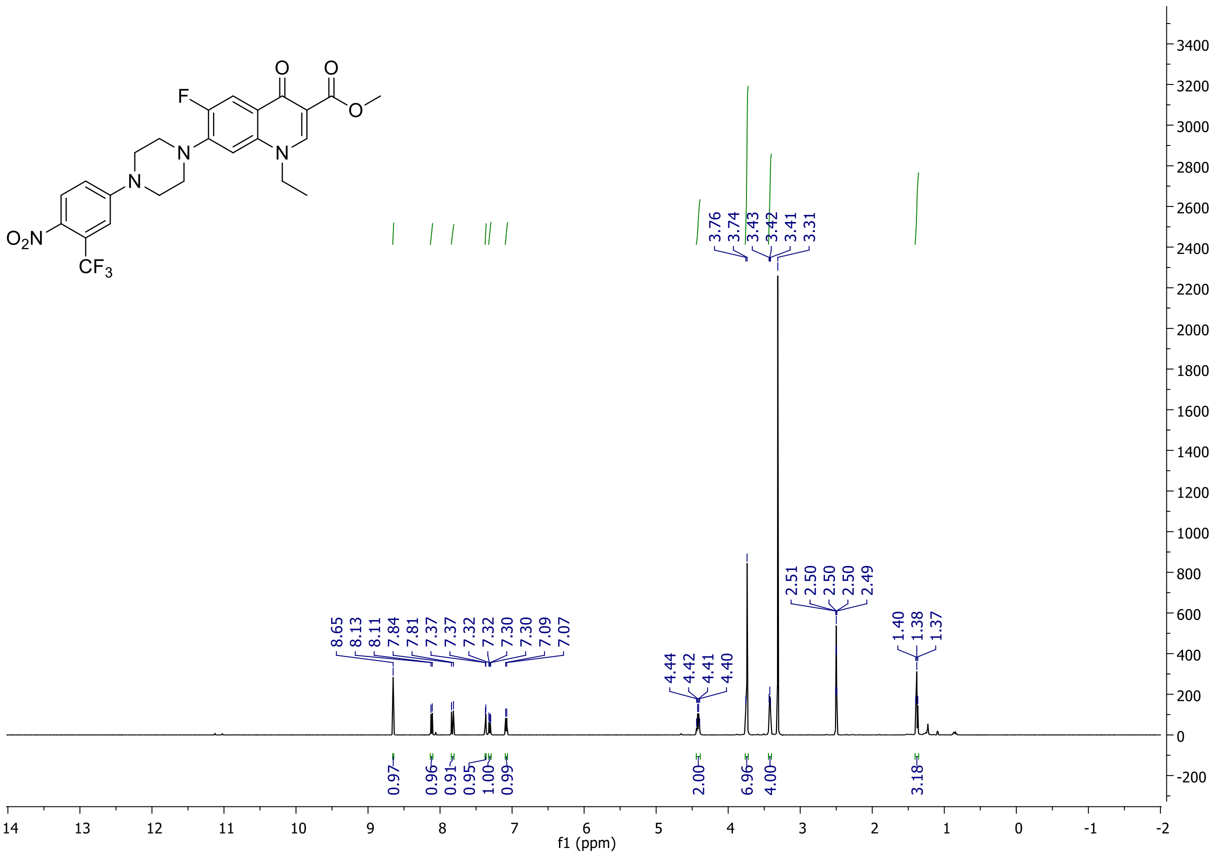

Figure S7. ^{1}H NMR (500 MHz, DMSO-*d6*) of compound **3b**.

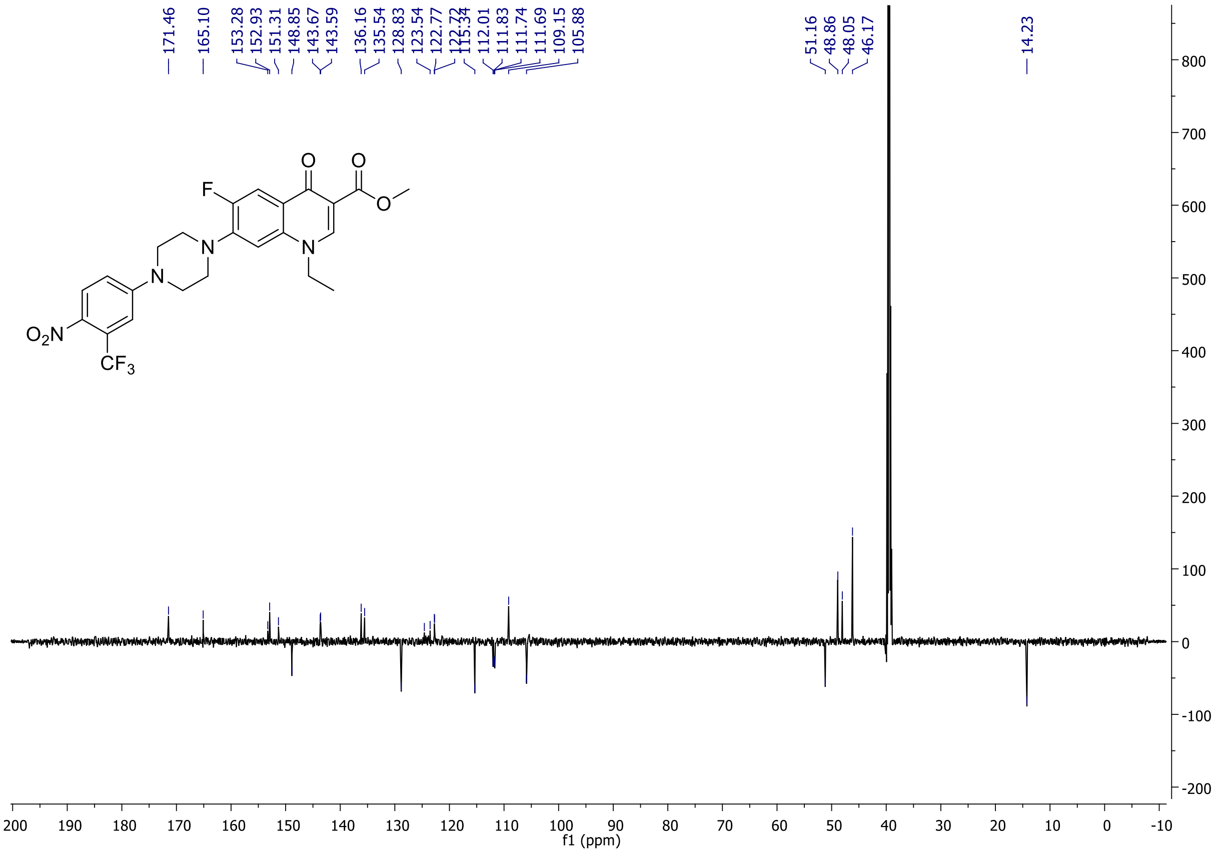

Figure S8. ^{13}C NMR (125 MHz, DMSO-*d6*) of compound **3b**.

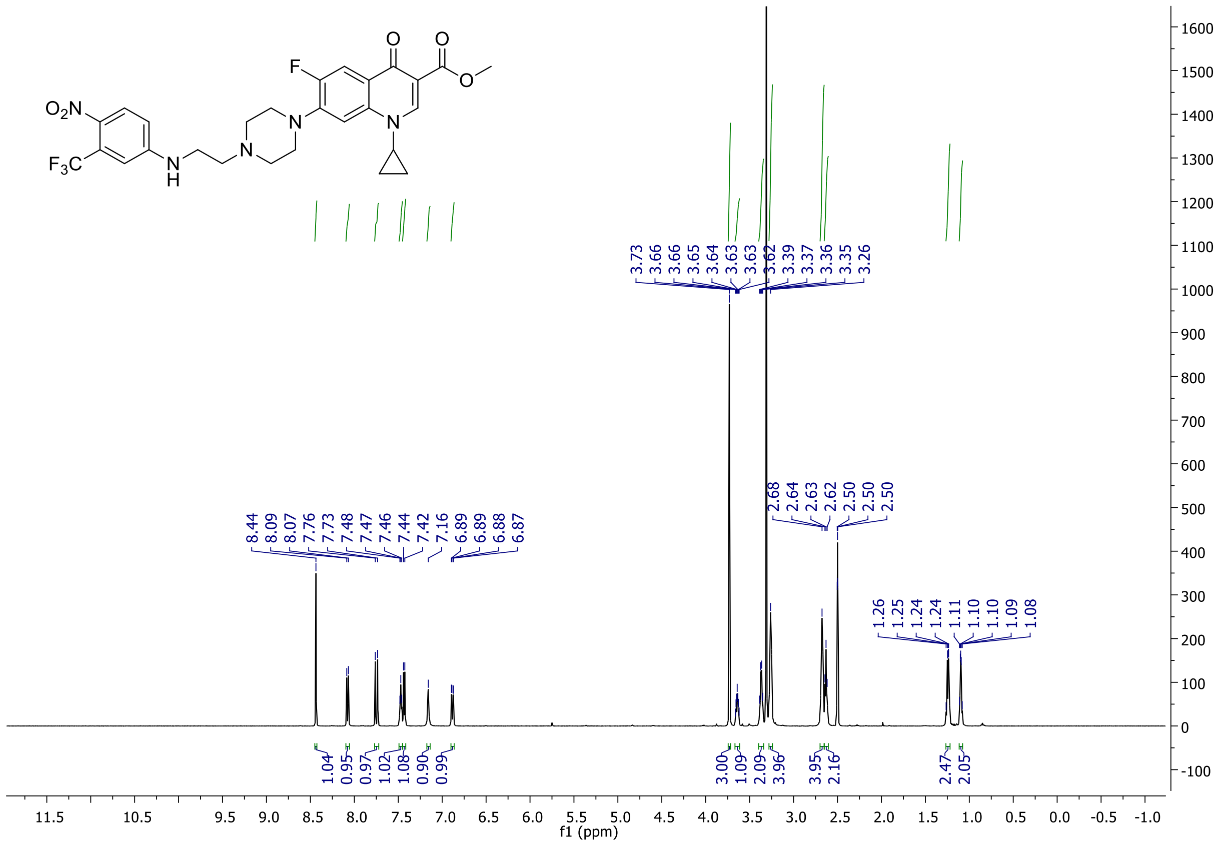

Figure S9. ^{1}H NMR (500 MHz, DMSO-*d6*) of compound **6a**.

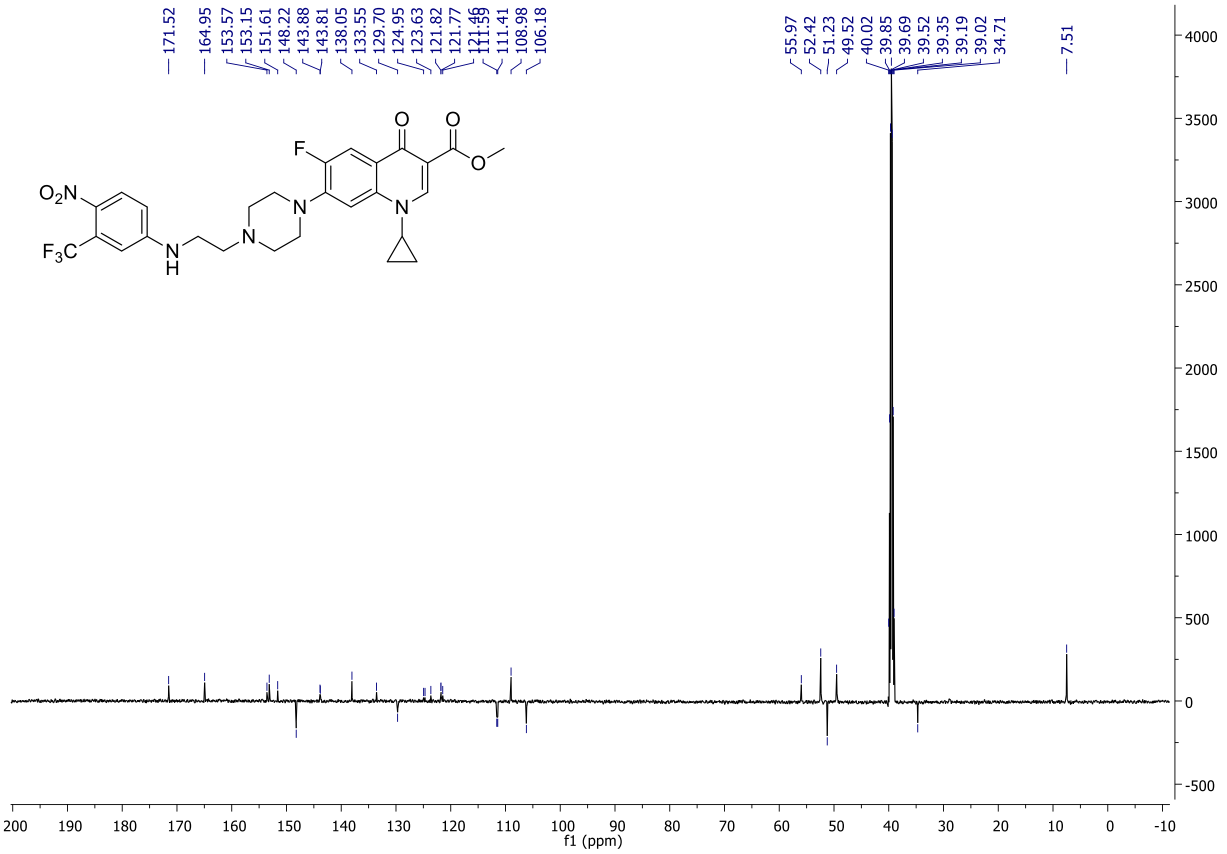

Figure S10. ^{13}C NMR (125 MHz, DMSO-*d6*) of compound **6a**.

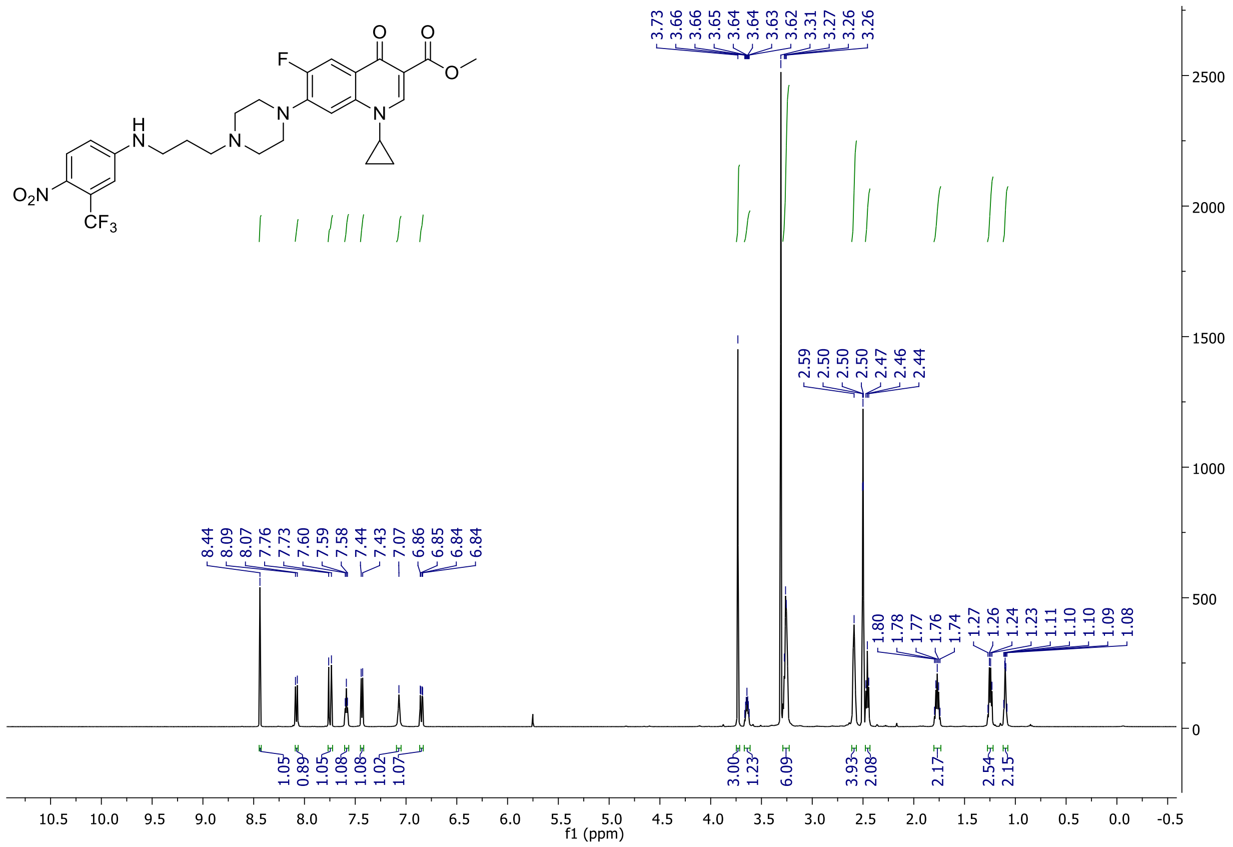

Figure S11. ^{1}H NMR (500 MHz, DMSO-*d6*) of compound **6b**.

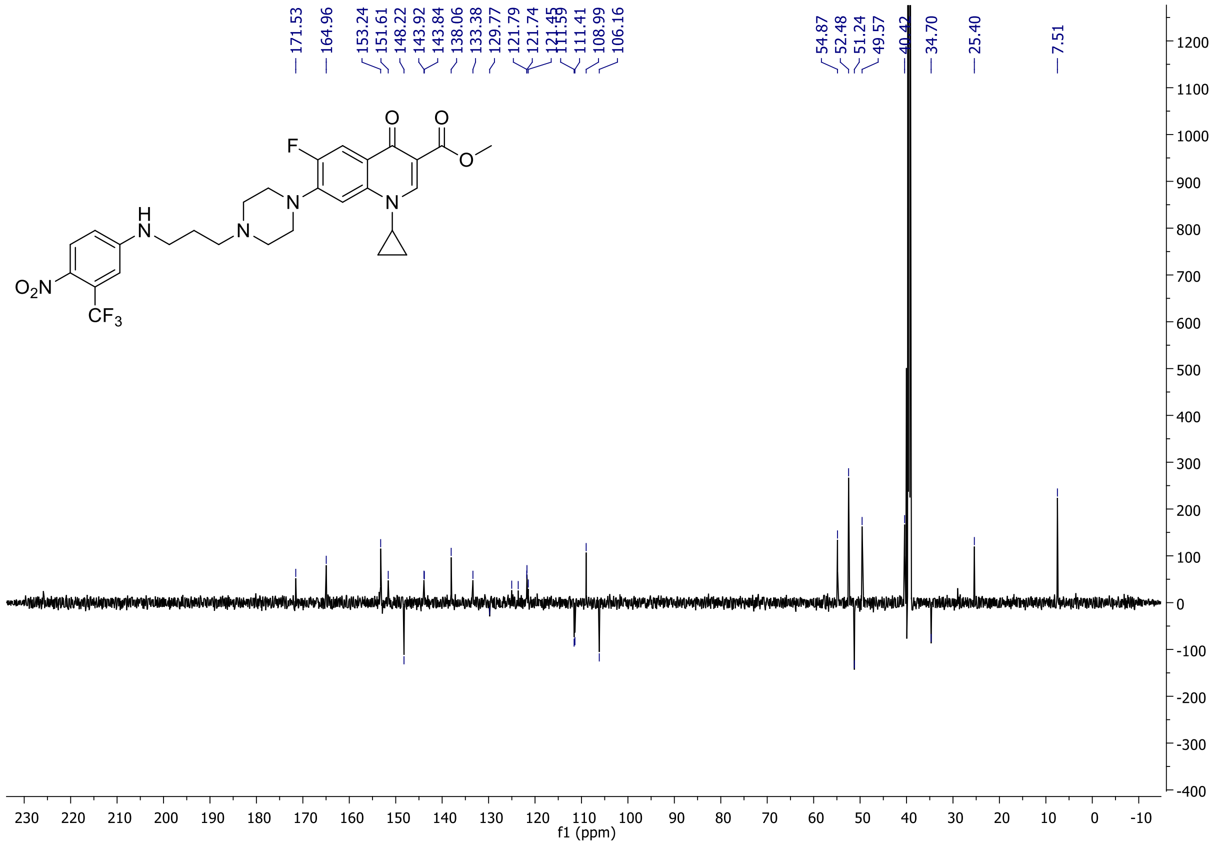

Figure S12. ^{13}C NMR (125 MHz, DMSO-*d6*) of compound **6b**.

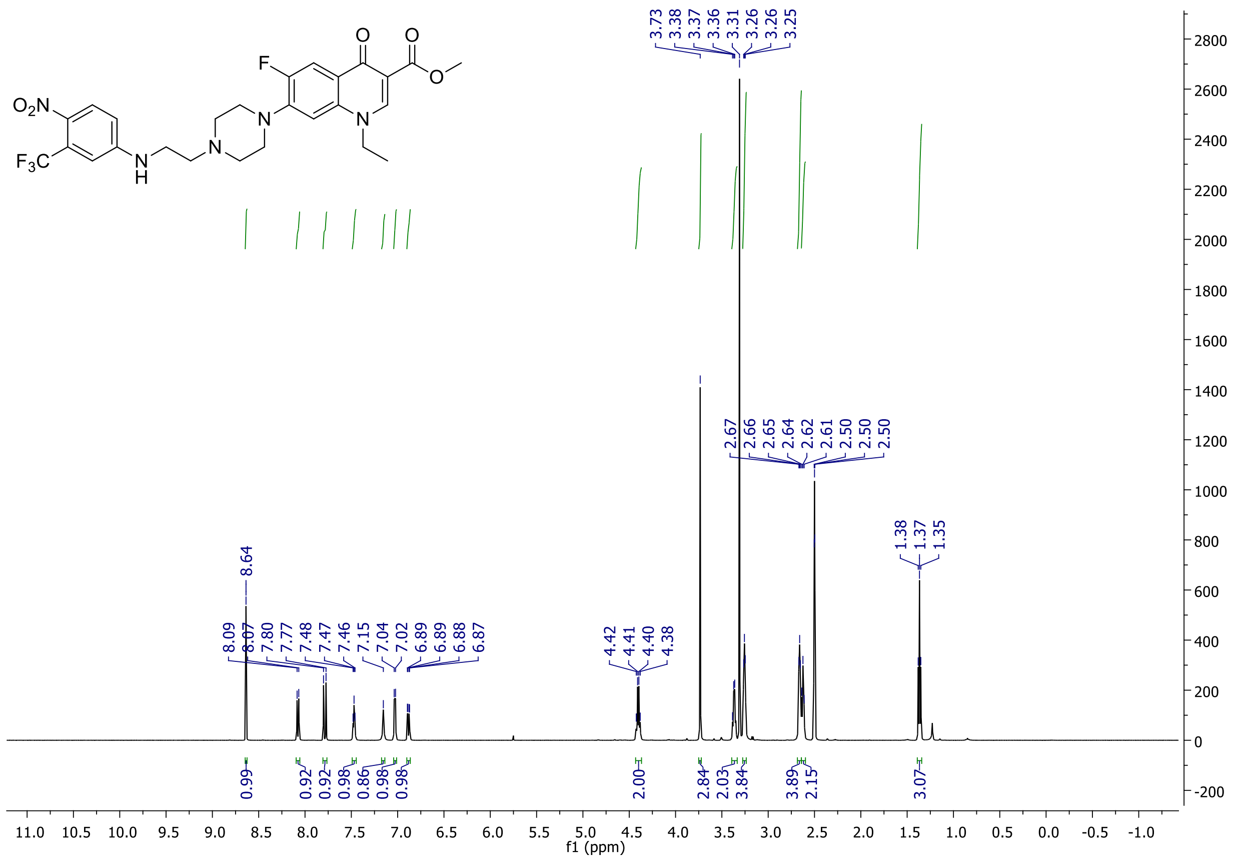

Figure S13. ^{1}H NMR (500 MHz, DMSO-*d6*) of compound **6c**.

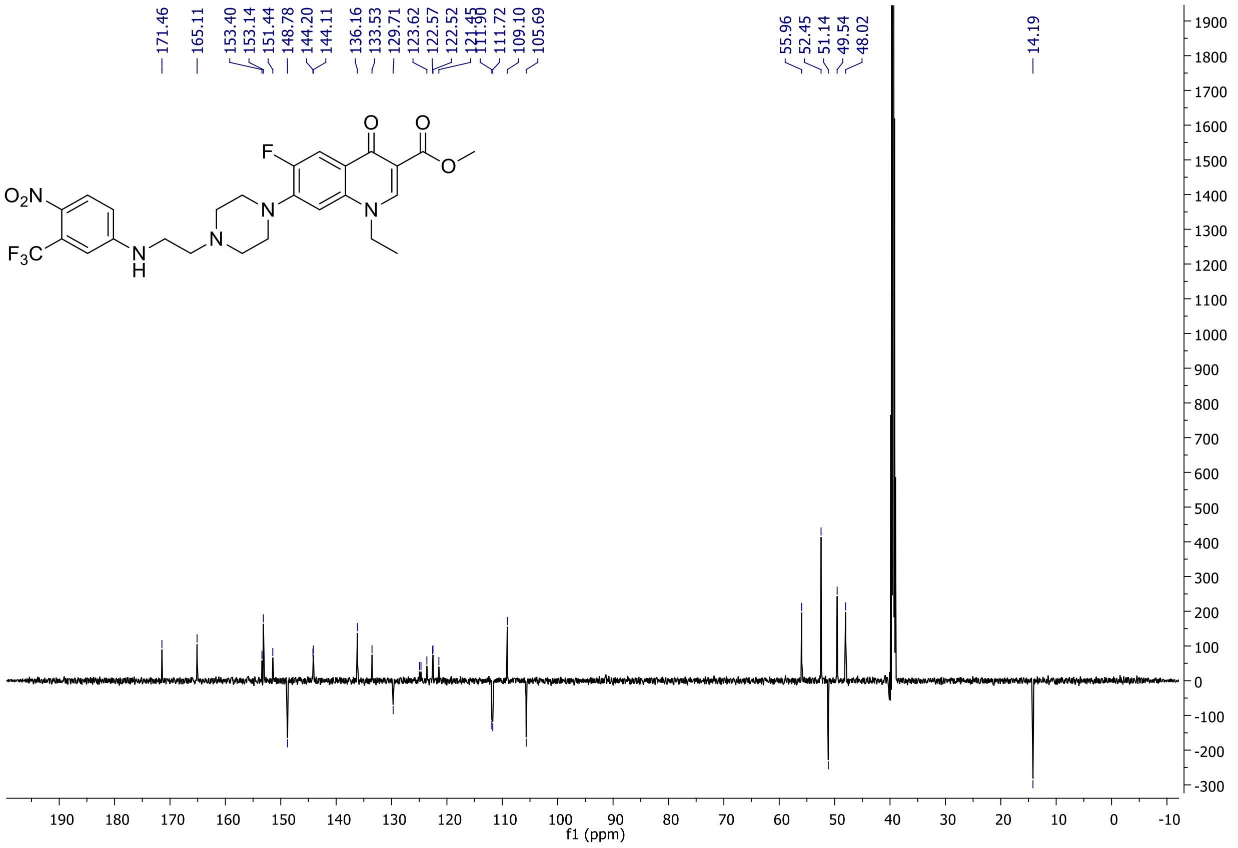

Figure S14. ^{13}C NMR (125 MHz, DMSO-*d6*) of compound **6c**.

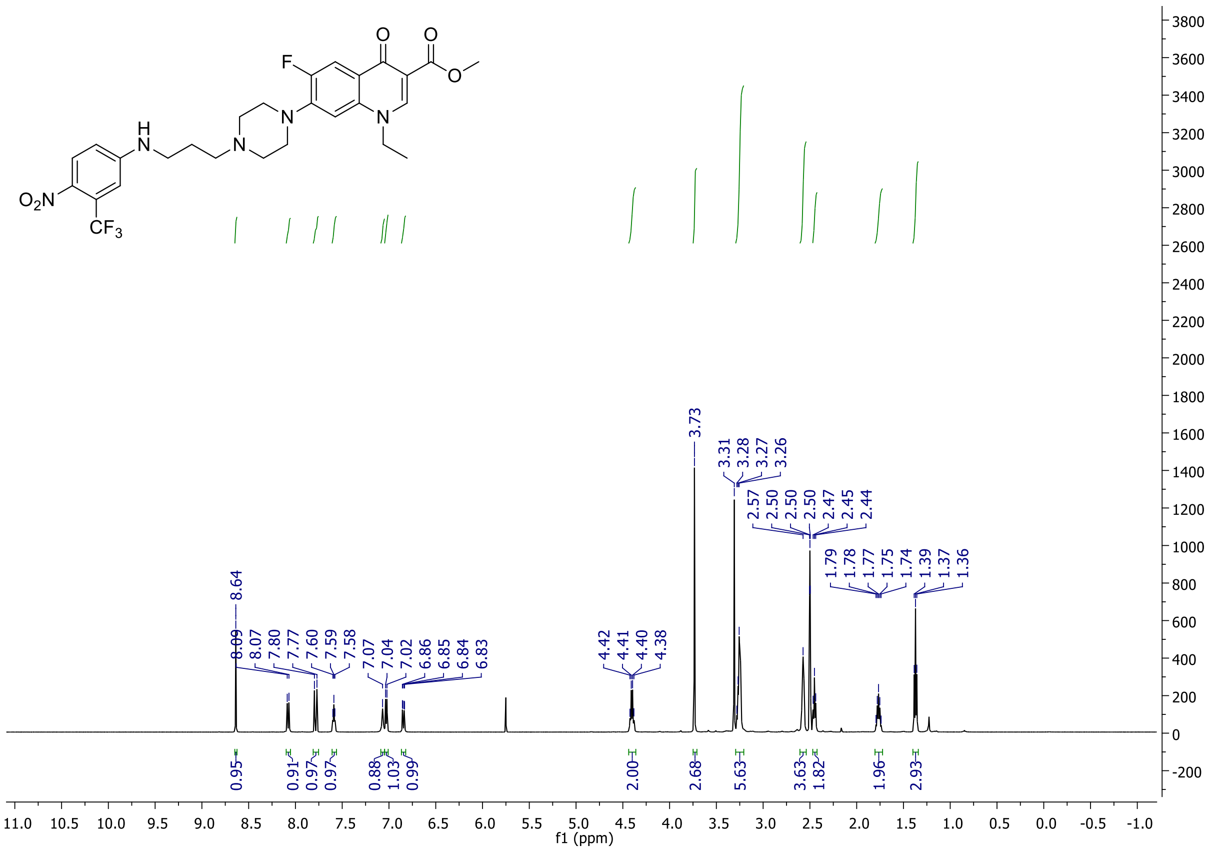

Figure S15. ^{1}H NMR (500 MHz, DMSO-*d6*) of compound **6d**.

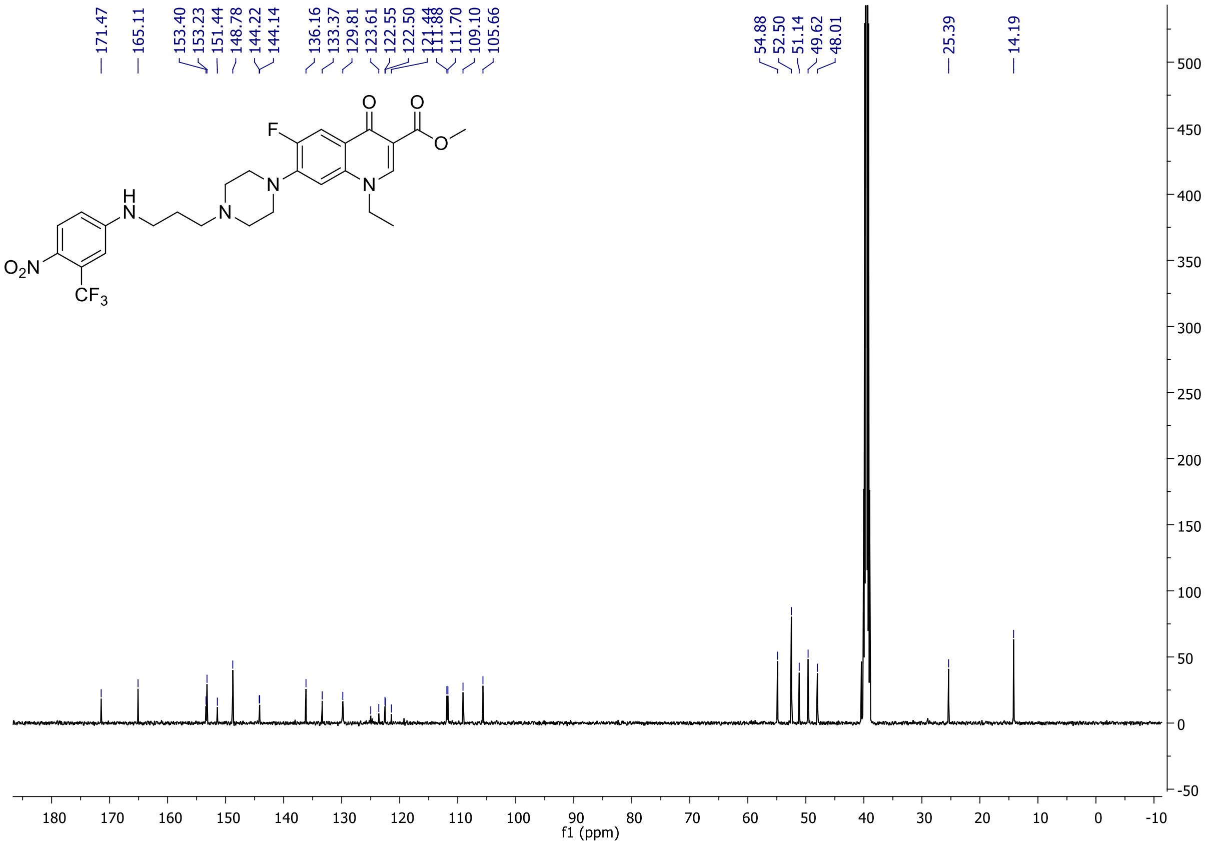

Figure S16. ^{13}C NMR (125 MHz, DMSO-*d6*) of compound **6d**.

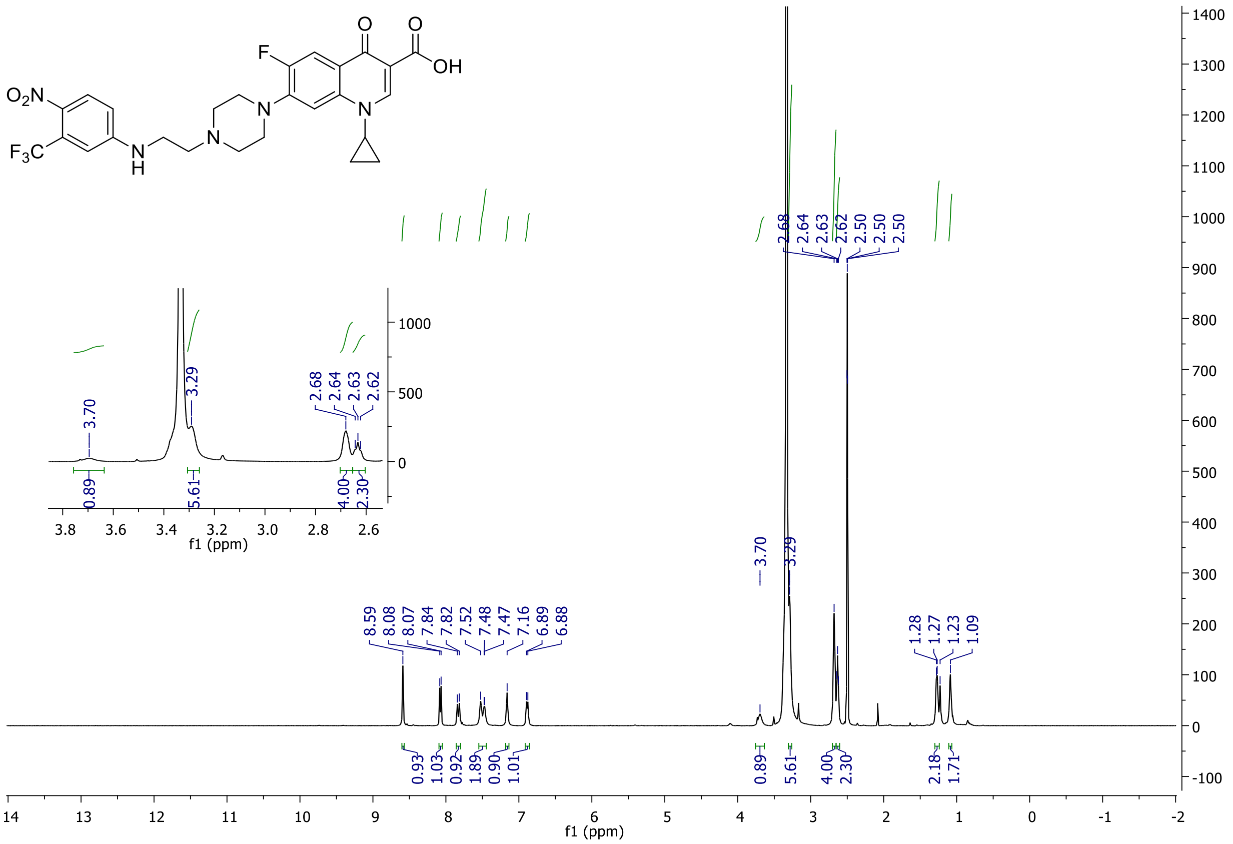

Figure S17. ^{1}H NMR (500 MHz, DMSO-*d6*) of compound **7a**.

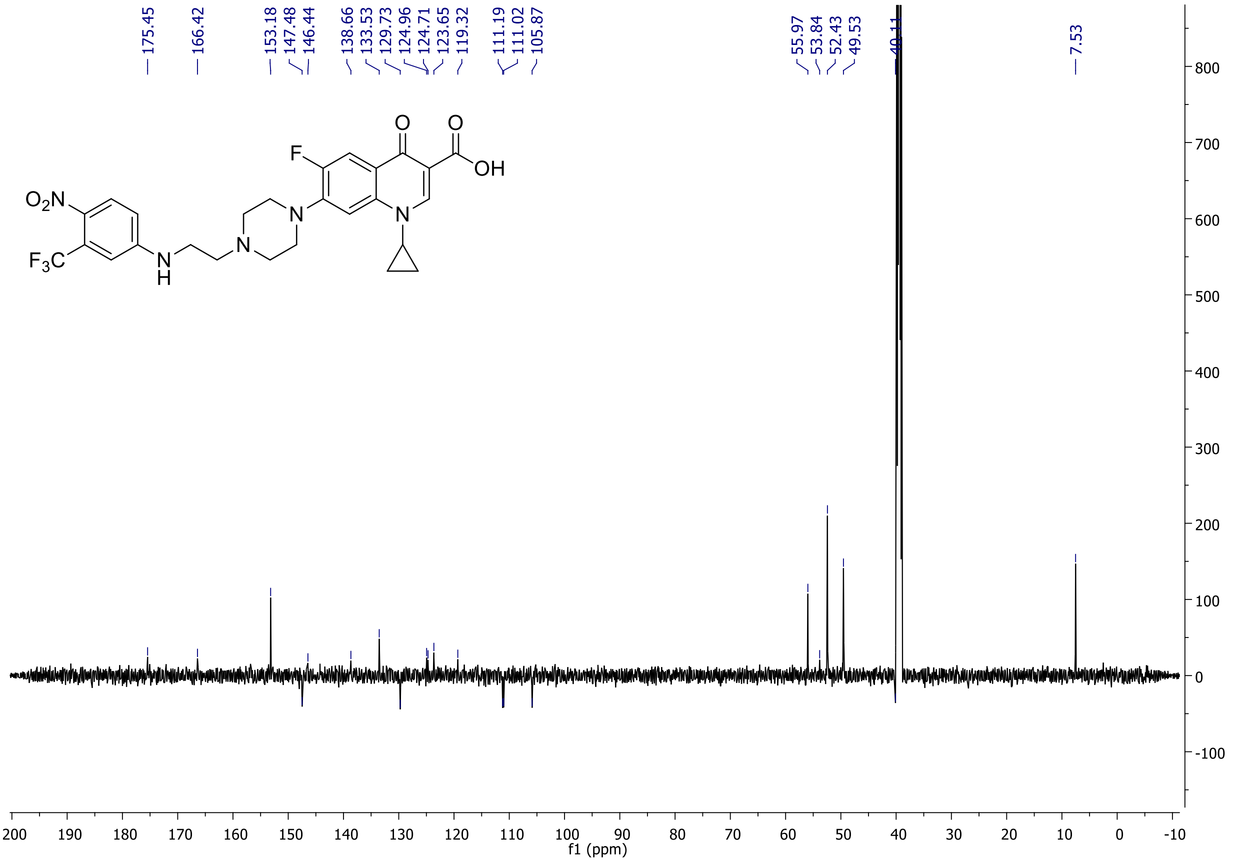

Figure S18. ^{13}C NMR (125 MHz, DMSO-*d6*) of compound **7a**.

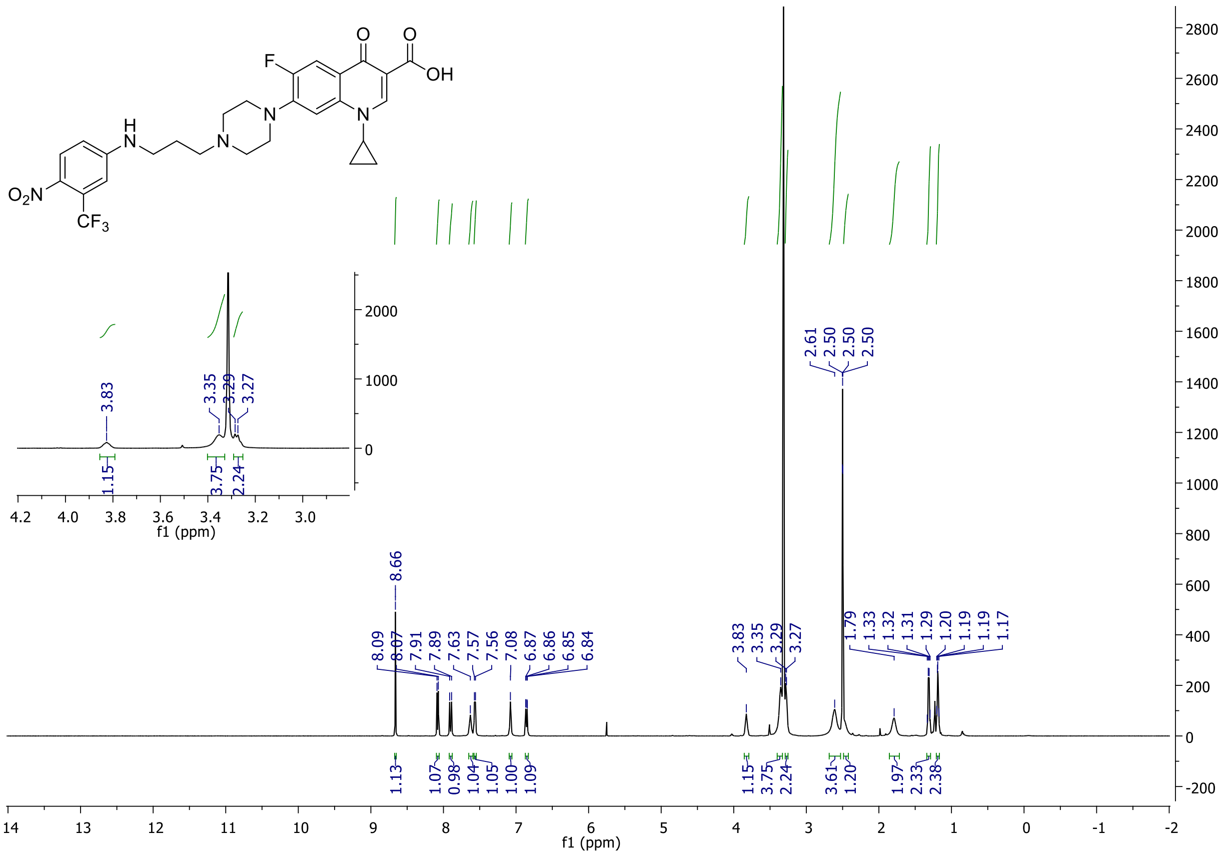

Figure S19. ^{1}H NMR (500 MHz, DMSO-*d6*) of compound **7b**.

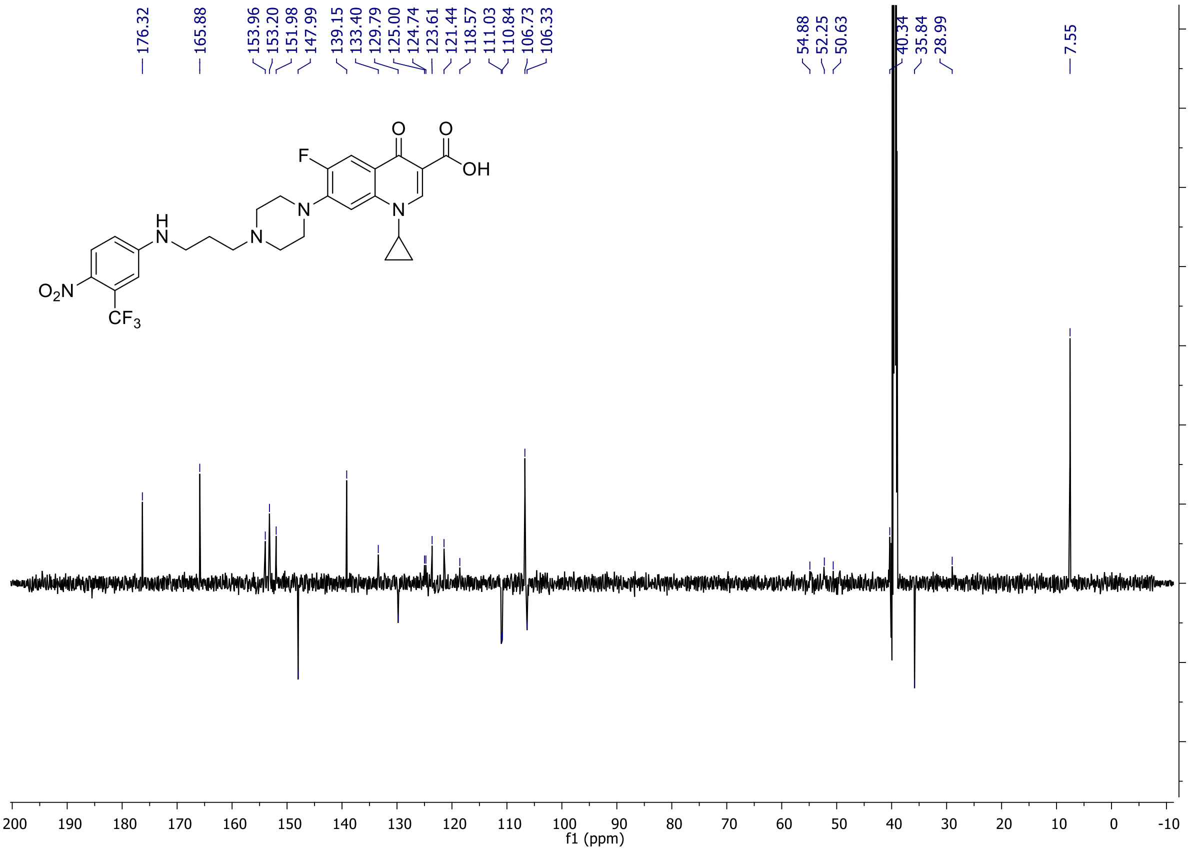

Figure S20. ^{13}C NMR (125 MHz, DMSO-*d6*) of compound **7b**.

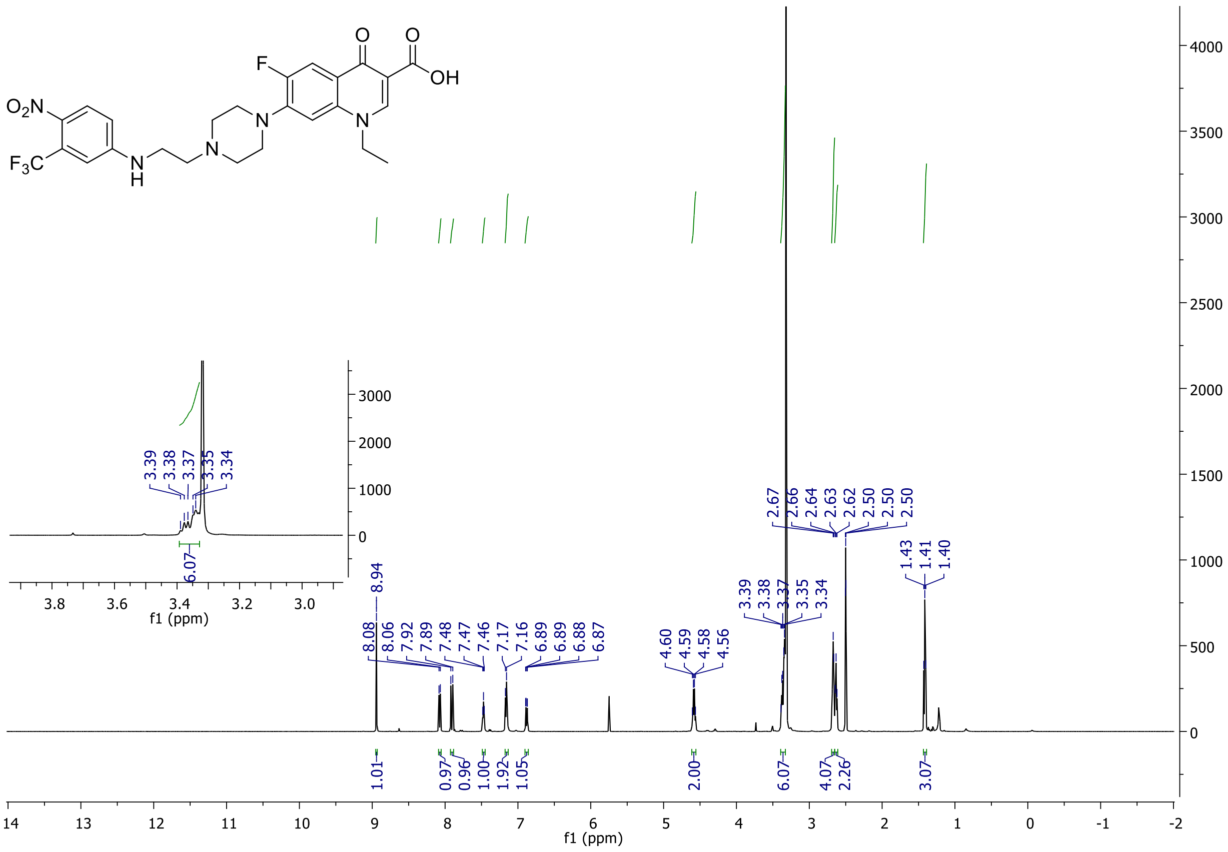

Figure S21. ^{1}H NMR (500 MHz, DMSO-*d6*) of compound **7c**.

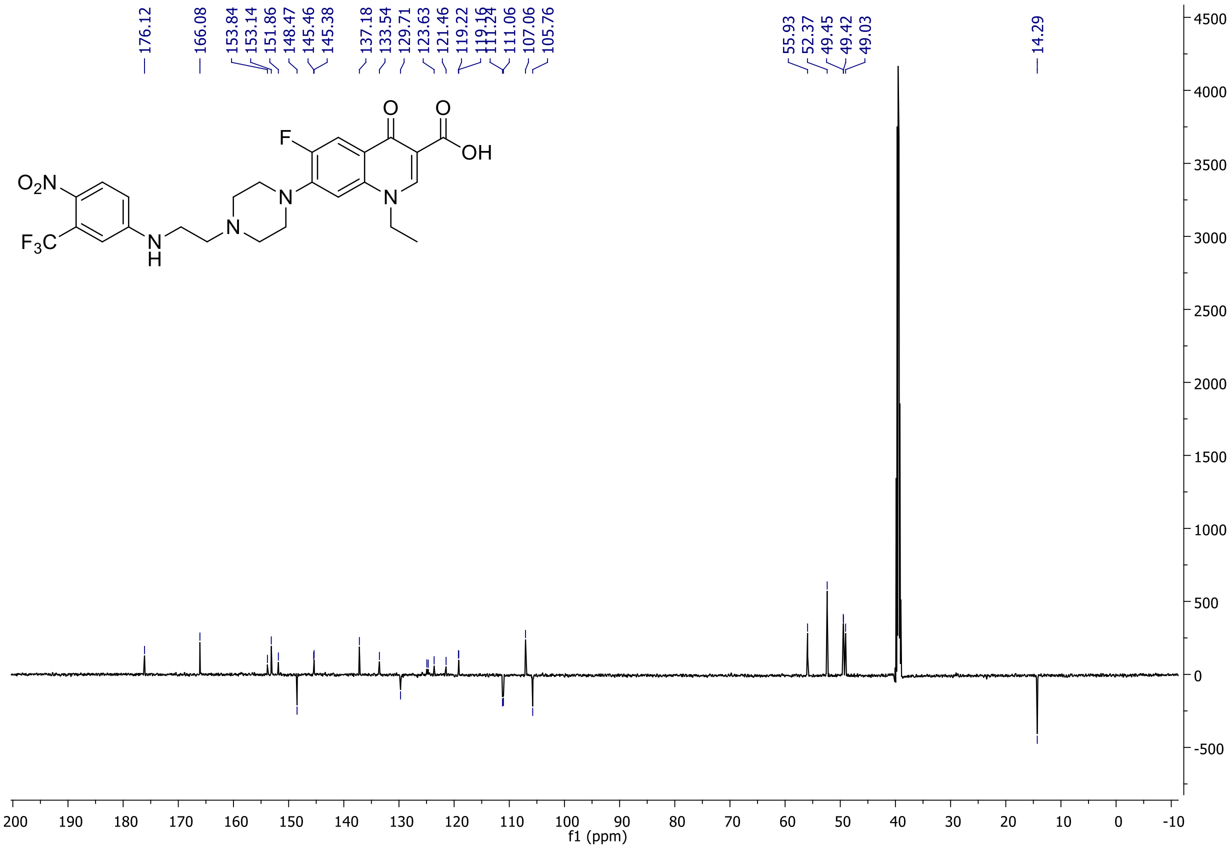

Figure S22. ^{13}C NMR (125 MHz, DMSO-*d6*) of compound **7c**.

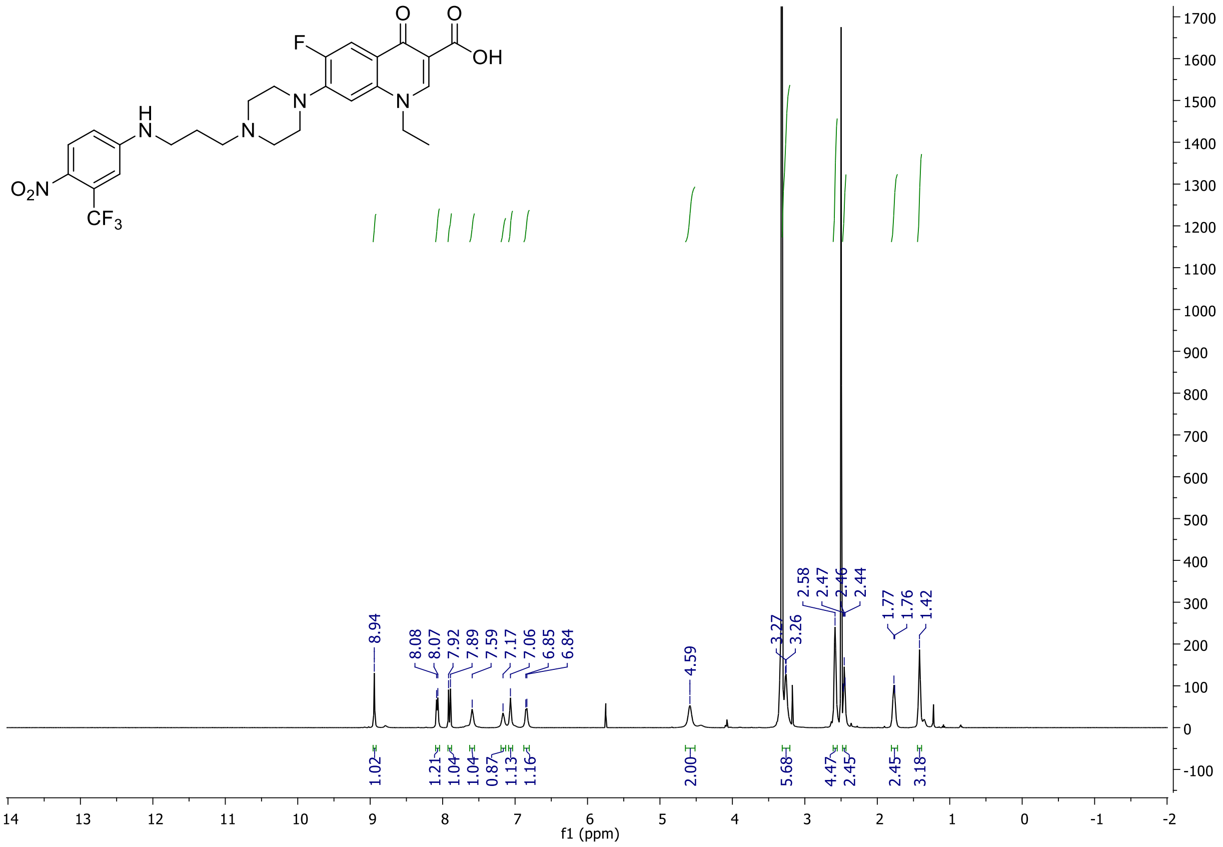

Figure S23. ^{1}H NMR (500 MHz, DMSO-*d6*) of compound **7d**.

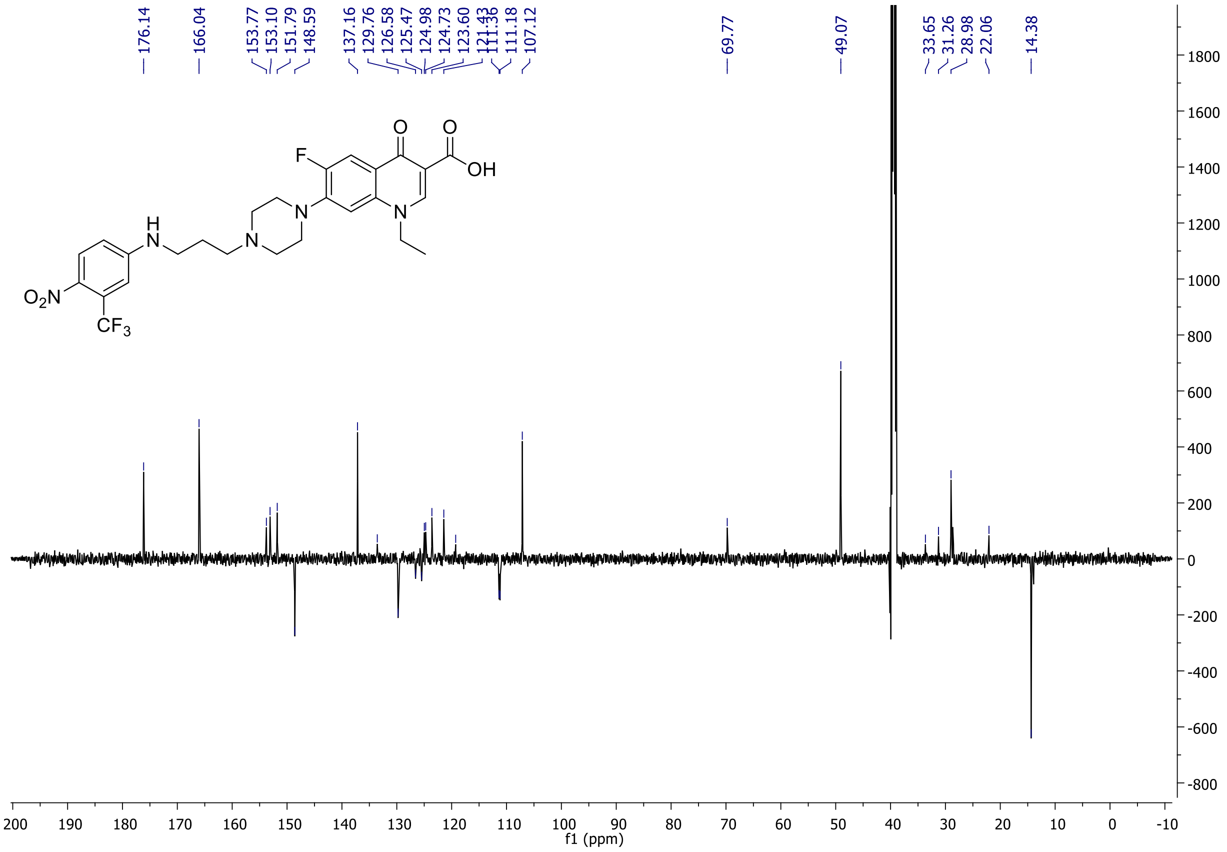

Figure S24. ^{13}C NMR (125 MHz, DMSO-*d6*) of compound **7d**.

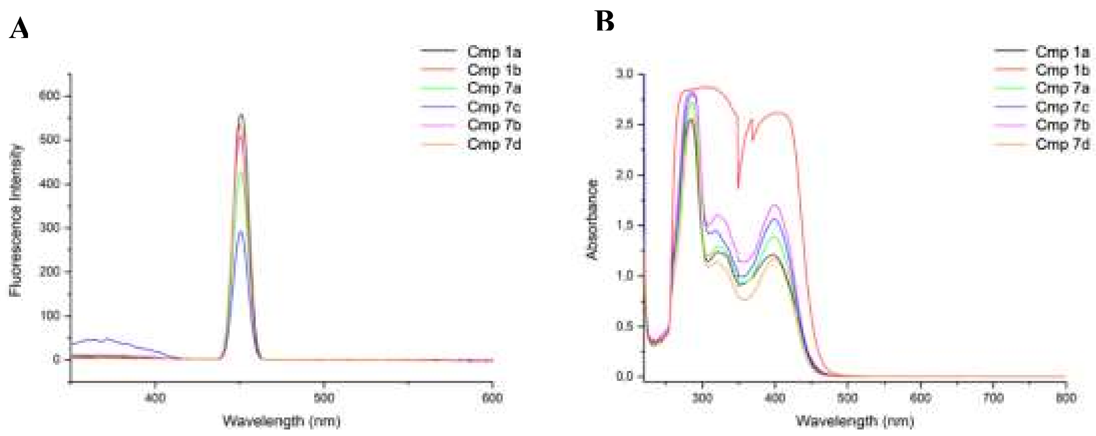

Figure S25. **A)** Fluorescence spectrum of **1a**,**b**, and **7a**–**d** (5 μM DMSO); **B)** Absorption spectrum of **1a**,**b**, and **7a**–**d** (50 μM DMSO).

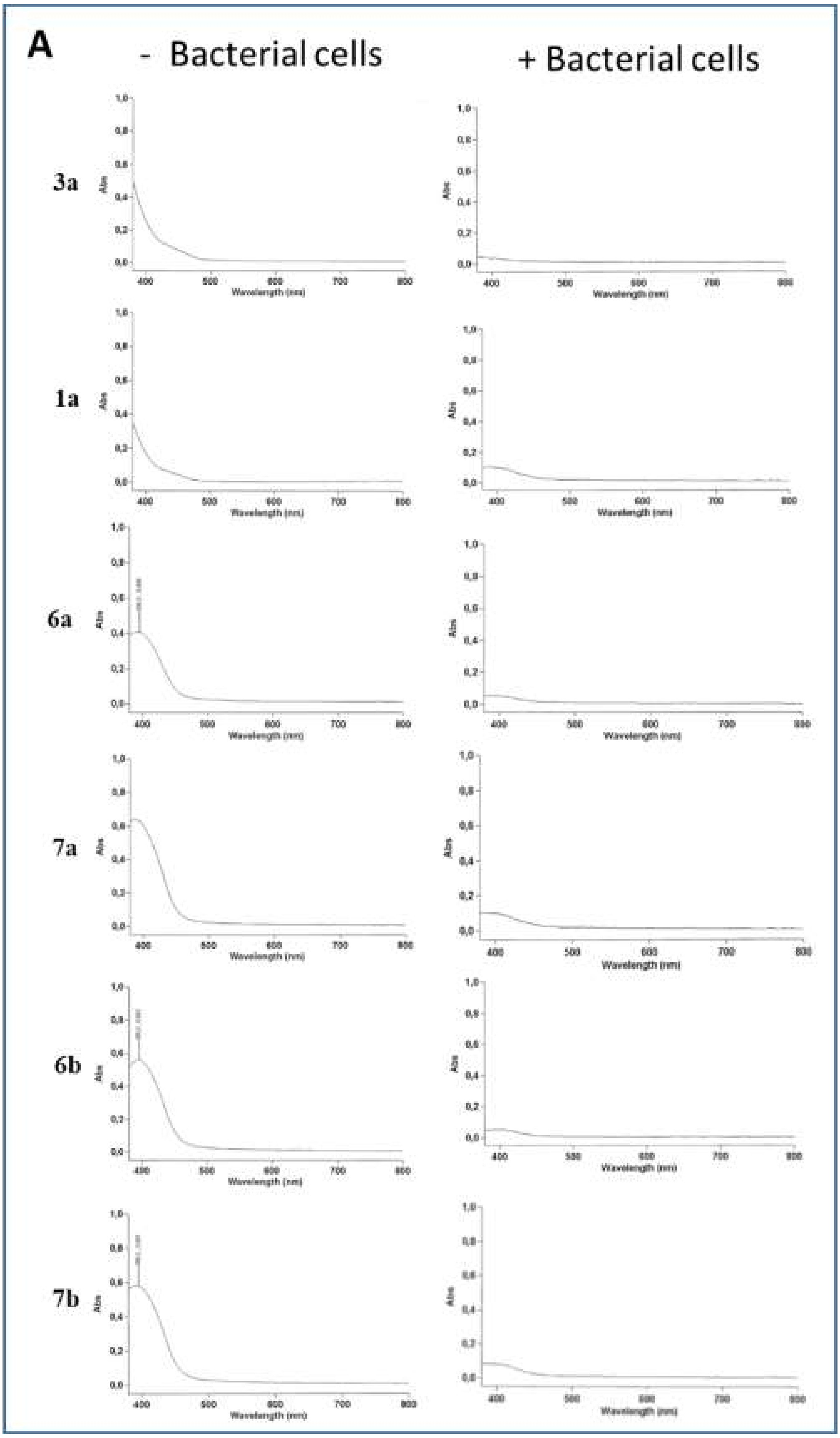

Figure S26. Visible spectrum of Ciprofloxacin derivatives administered at 10 μM, without (first column) and with cells (second column).

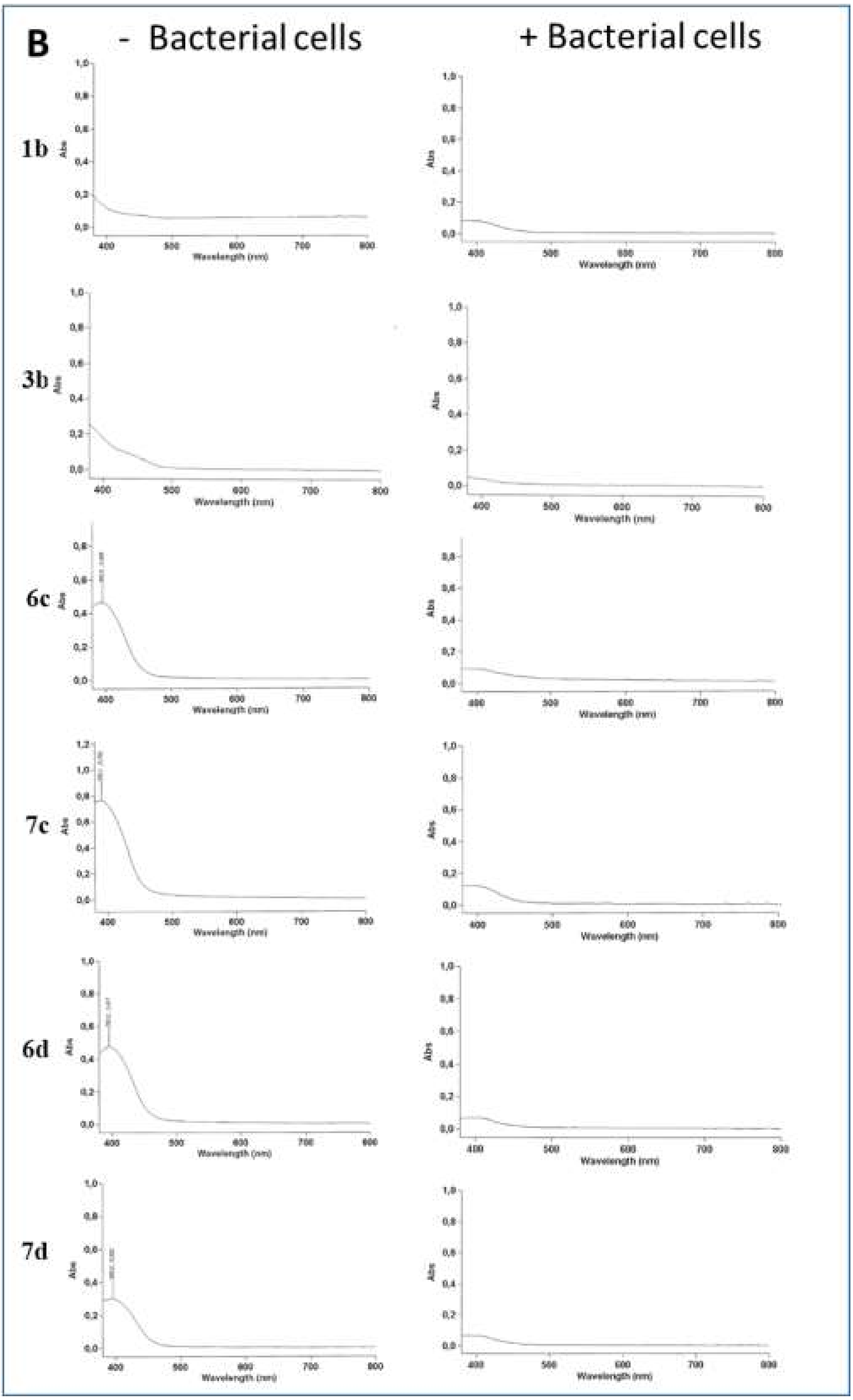

Figure S27. Visible spectrum of Norfloxacin derivatives administered at 10 µM, without (first column) and with cells (second column).

Table S1. IC_{50} Values of Compounds 1a, 3a, 6a,b and 7a,b on DU145, PC3, MCF7, MDA-MB231 and HCT116 Cell Lines

	IC_{50} (μM) ± ES[a]				
Compd	DU145	PC3	MCF7	MDA-MB231	HCT116
**1a**	7.78 ±1.52	6.45 ±0.19	8.80 ±1.59	8.06 ±1.05	9.67 ±0.36
**3a**	5.67 ±0.06	15.92 ±0.50	15.45 ±1.20	10.72 ±1.90	6.93 ±1.53
**6a**	4.80 ±0.96	6.72 ±0.35	16.84 ±2.40	16.08 ±2.10	15.70 ±2.00
**6b**	14.03 ±1.40	3.17 ±0.47	12.12 ±1.10	3.76 ±0.94	5.21 ±0.73
**7a**	2.56 ±0.21	2.48 ±0.42	3.12 ±0.62	2.24 ±0.18	5.29 ±0.72
**7b**	2.42 ±0.90	3.02 ±0.59	5.23 ±0.82	2.51 ±0.43	1.83 ±0.01
Cip	24.88 ±0.80	33.90 ±2.90	8.85 ±0.09	19.88 ±3.60	31.47 ±1.50

[a]Mean ± ES 4/5 independent experiments.

Table S2. IC_{50} Values of Compounds 1b, 3b, 6c,d and 7c,d on DU145, PC3, MCF7, MDA-MB231 and HCT116 Cell Lines

	IC_{50} (μM) ± ES[a]				
Compd	DU145	PC3	MCF7	MDA-MB231	HCT116
**1b**	2.85 ±0.01	3.07 ±0.64	5.24 ±0.70	6.93 ±0.85	10.38 ±1.50
**3b**	6.06 ±0.51	15.32 ±1.20	11.68 ±1.40	10.16 ±3.10	9.73 ±2.25
**6c**	4.77 ±0.99	9.26 ±1.29	15.04 ±0.10	11.89 ±1.00	12.40 ±2.90
**6d**	5.49 ±0.97	4.10 ±0.61	10.53 ±1.20	8.89 ±1.65	11.66 ±2.10
**7c**	1.57 ±0.59	2.33 ±0.02	2.27 ±0.31	1.52 ±0.40	2.15 ±0.39
**7d**	1.56 ±0.19	4.83 ±1.15	5.83 ±0.82	3.64 ±0.63	5.36 ±0.65
Nor	12.01 ±0.60	33.56 ±3.00	19.37 ±0.70	14.89 ±2.70	12.53 ±2.60

[a]Mean ± ES 4/5 independent experiments.

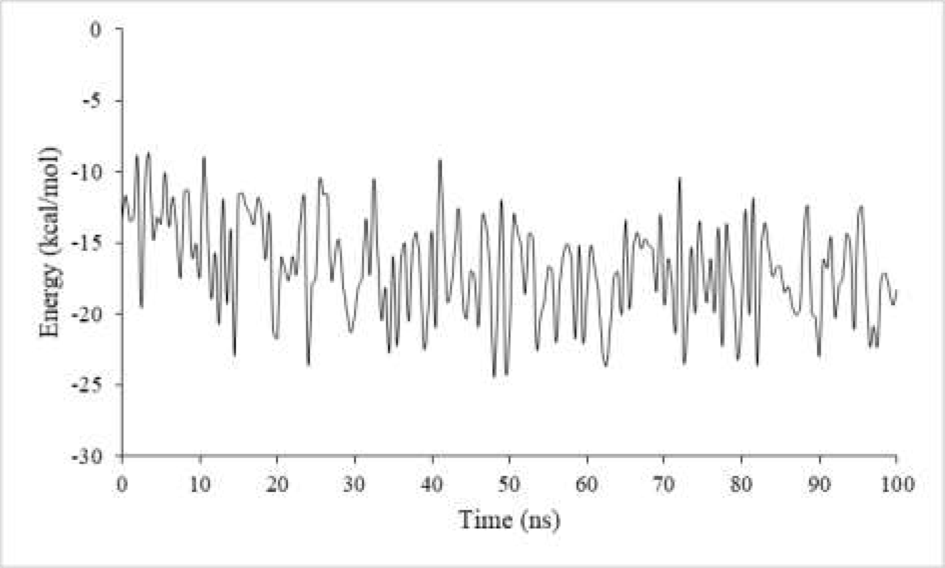

Figure S28. MM/PBSA **v**ariation of the energy of binding along the MD simulation trajectory for compound **1a**.
